# Exosomes as mediators of platinum resistance in ovarian cancer

**DOI:** 10.18632/oncotarget.14440

**Published:** 2017-01-02

**Authors:** Jennifer Crow, Safinur Atay, Samagya Banskota, Brittany Artale, Sarah Schmitt, Andrew K Godwin

**Affiliations:** ^1^ Department of Pathology and Laboratory Medicine, University of Kansas Medical Center, Kansas City, KS; ^2^ Department of Biomedical Engineering, Duke University, Durham, North Carolina, NC; ^3^ Bioengineering Graduate Program, University of Kansas, Lawrence, KS; ^4^ University of Kansas Cancer Center, Kansas City, KS

**Keywords:** ovarian cancer, EMT, exosomes, platinum-resistance, SMAD4

## Abstract

Exosomes have been implicated in the cell-cell transfer of oncogenic proteins and genetic material. We speculated this may be one mechanism by which an intrinsically platinum-resistant population of epithelial ovarian cancer (EOC) cells imparts its influence on surrounding tumor cells. To explore this possibility we utilized a platinum-sensitive cell line, A2780 and exosomes derived from its resistant subclones, and an unselected, platinum-resistant EOC line, OVCAR10. A2780 cells demonstrate a ~2-fold increase in viability upon treatment with carboplatin when pre-exposed to exosomes from platinum-resistant cells as compared to controls. This coincided with increased epithelial to mesenchymal transition (EMT). DNA sequencing of EOC cell lines revealed previously unreported somatic mutations in the *Mothers Against Decapentaplegic Homolog 4* (*SMAD4*) within platinum-resistant cells. A2780 cells engineered to exogenously express these *SMAD4* mutations demonstrate up-regulation of EMT markers following carboplatin treatment, are more resistant to carboplatin, and release exosomes which impart a ~1.7-fold increase in resistance in naive A2780 recipient cells as compared to controls. These studies provide the first evidence that acquired *SMAD4* mutations enhance the chemo-resistance profile of EOC and present a novel mechanism in which exchange of tumor-derived exosomes perpetuates an EMT phenotype, leading to the development of subpopulations of platinum-refractory cells.

## INTRODUCTION

Ovarian cancer remains the leading cause of death of all gynecological malignancies due, in part, to a dearth of treatment options and the evolution of platinum-resistant disease. The clinical introduction and use of platinum–based chemotherapeutics, specifically *cis*-diamminedichloroplatinum(II) (cisplatin), significantly improved overall survival (OS) by ~6 months in 29% of patients with ovarian cancer, leading the way to its adoption as the backbone of most chemotherapeutic regimens [1, 2]. In the mid-1980’s, a cisplatin analog, Carboplatin [cis-diammine(1,1-cyclobutanedicarboxylato)platinum(II)], with an improved toxicity profile and equivalent therapeutic efficacy, replaced cisplatin as standard of care [3–5]. The last major advance occurred in the early 1990's with the introduction of the mitotic inhibitor, paclitaxel, that further improved OS 3-15 months (depending on the study) when used in combination with platinum [6–8]. Despite these advances, tumor progression and recurrences still occur in approximately 80% of advanced stage ovarian cancer patients and ultimately result in mortality as a result of acquired resistance to platinum-based antineoplastic drugs. Platinum-resistance has been attributed to several factors including, but not limited to, increased glutathione synthesis, increased drug efflux, increased DNA damage repair, and/or increased ability to undergo epithelial to mesenchymal transition (EMT) [9–15].

Current interest in the EOC field is focused on the importance of intercellular cross talk mediated by soluble and insoluble factors between the EOC tumor and stromal cells during development, progression, and evolution of drug-resistance [16–18]. The EOC tumor microenvironment includes recruited host cells (*i.e*., endothelial cells, fibroblasts, and macrophages) that communicate with tumor cells and often are re-educated to supply functions which enhance metastasis, vascularization, and immuno-evasion. For instance, EOC tumors have been shown to produce significant levels of interleukin (IL)-6 which trigger recruitment of monocytes from the peripheral blood, and via activation of STAT3, convert these cells into tumor supportive M2 tumor associated macrophages [19]. Work from our lab demonstrated fibroblasts from normal human ovaries secrete high levels of hepatocyte growth factor, (HGF) which binds to and activate c-MET mediated signaling on EOC tumor cells. This interaction leads to changes in biological processes including increases in tumor proliferation and metastasis [20]. The mechanisms of cellular communication in these types of experiments include secretion of soluble factors such as cytokines, mitogens, and growth factors; however, recently nano-sized vesicles (30-150 nm) termed exosomes have been shown to be released by tumor cells and are emerging as a novel vehicle of cell-cell communication within the development and progression of EOC [21, 22].

Exosomes are multifunctional vesicles that have essential roles in normal physiology [23] as well as pathological processes [24–26]. They are formed through the endosomal pathway when the membrane of the late endosome undergoes inward budding resulting in the formation of intraluminal vesicles. The late endosome, now termed a multivesicular body (MVB), escapes merging with the lysosomal pathway and instead travels back and fuses with the cell membrane, releasing its contents (exosomes) into the extracellular space [27]. Exosomes are informative molecules that carry cargo representative of their originating cell including nucleic acids, cytokines, membrane-bound receptors, and a wide assortment of other biologically active lipids and proteins [28, 29]. This cargo remains functional upon entry or fusion with a recipient cell, thus exosomal transfer is now considered an important form of cell-cell communication in normal and pathological states such as cancer [30]. Exosomes can travel systemically throughout the body and are able to target cells at very distal locations [31]. They contribute to EOC development by inducing immune evasion, assisting in the establishment of secondary tumor niches, and serve as ‘packaged delivery vehicles’ for cross talk between tumor cells and the surrounding stroma [32, 33]. More recently, exosomes have been shown to have a functional role in the development of chemotherapy resistance in breast, non-small cell lung, and prostate cancer; however, their role in platinum-resistance in EOC is unknown [34–36]. While emphasis in the EOC field is focused on tumor-stroma communication during neoplastic advancement, we report here, the first evidence of the importance of the intricate exchange of exosome-mediated crosstalk within EOC tumors leading to chemotherapeutic resistance by way of activation of EMT. Based on these novel findings, we propose the release of exosomes is a mechanism by which neoplastic EOC cells ‘educate’ each other, thereby exasperating the development of platinum-resistant disease.

## RESULTS

### Exosomes from carboplatin-resistant cells modify the platinum sensitivity profile of recipient cells

**Figure 1:**
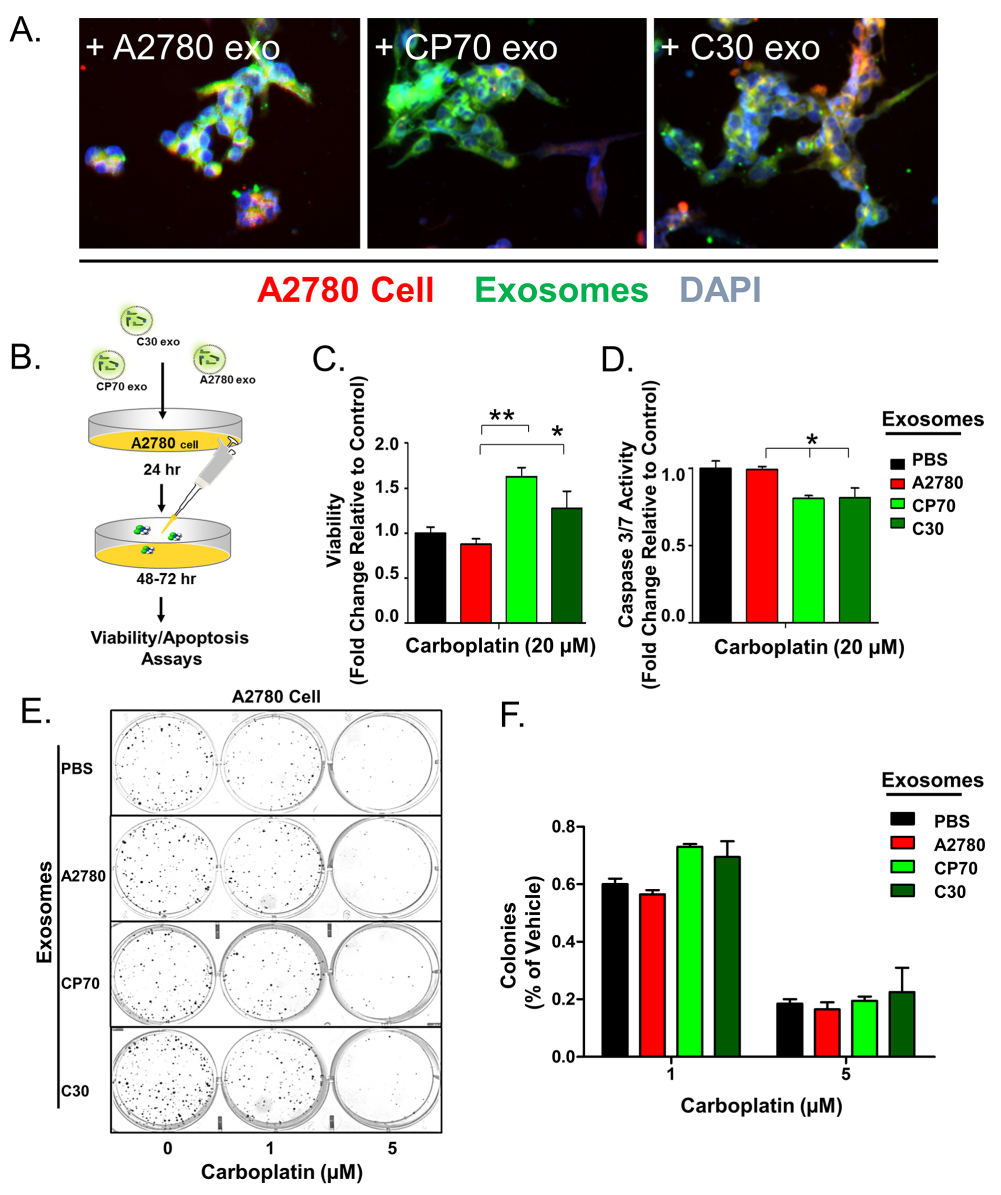
Exosome Transfer Corresponds with a Loss of Sensitivity to Platinum **A**. Overlay of A2780 cells (Red) exposed to exosomes (Green) derived from A2780, CP70, or C30 cell lines for 24 hours. DAPI (Blue) is included. **B**. Illustration of experimental protocol. **C**. Viability of A2780 cells pre-treated with PBS (Black), or exosomes derived from A2780 (Red), CP70 (Green), or C30 (Dark Green) cell lines followed by exposure to carboplatin. **D**. Caspase 3/7 activity as measured using Caspase Glo^®^ luminescent reporter system in A2780 cells exposed to the same conditions as C. **E-F**. Colony formation assay and corresponding analysis of 200 A2780 cells pre-treated for 24 hours with exosomes (as above) and then exposed to constant levels of 0, 1, or 5 μM carboplatin. All values are normalized to vehicle. Fold changes are relative to control (PBS – no exosome group) *P<0.05, **P<0.01.

Exosomes carry a range of nucleic acids and proteins and can have a significant impact on the phenotype of recipient cells as reported in melanoma, breast, non-small cell lung, and gastrointestinal stromal tumors (GIST) amongst others [35, 37–40]. For this phenotypic effect to occur, exosomes need to fuse with target cell membranes, either directly with the plasma membrane or with the endosomal membrane after endocytic uptake. To examine if exosomes isolated from platinum-resistant clones are taken up by the platinum-sensitive parental, population we utilized the classic A2780 (IC_50_ of 11 μM carboplatin) cell line and two independently-derived carboplatin resistant clones, C30 (IC_50_ of 325 μM carboplatin) and CP70 (IC_50_ of 120 μM carboplatin) [41–43] (Supplementary Table 1). We first isolated extracellular vesicles from conditioned media of A2780, CP70, and C30 cells by ultracentrifugation [44, 45]. Isolated vesicles displayed between ~80-150 nm in size as determined by Nanoparticle Tracking Analysis (NTA) and scanning electron microscopy and contained common exosomal markers such as ALIX, TSG101, CD60, and the absence of either β-actin or GRP78 suggesting a highly enriched exosome population (Supplementary Figure 1). To visualize uptake of exosomes by recipient cells, the exosomes were labeled with PKH67 green and the cells with PKH red fluorescent membrane dyes. Recipient A2780 cells were then exposed to exosomes (number of exosome equivalent to 1 μg of total exosomal protein/10,000 cells) derived from A2780 (autologous), C30, or CP70 cells for up to 24 hours. Regardless of the source, exosome uptake was rapid and uniform across all experimental groups (Figure 1A, Supplementary Figures 2-4).

We have previously shown that the phenotypes of normal primary myometrial cells are dramatically altered towards the characteristics of donor tumor cells following uptake of tumor-derived exosomes from GIST cells and patient samples [38]. To investigate if a platinum-resistant phenotype could be transferred by this mechanism we treated platinum-naive A2780 cells with exosomes (number of exosome equivalent to 0.5 μg of total exosomal protein/5,000 cells) from A2780 (autologous), CP70, C30, or vehicle (PBS) for 24 hours prior to treatment with 20 μM carboplatin for 48 hours (Figure 1B). We observed a near 2-fold (P<0.05) and 1.5-fold increase in viability of A2780 cells exposed to CP70-derived and C30-derived exosomes, respectively, as compared to autologous exosomes or PBS (Figure 1C). Parallel analysis of caspase 3/7 activity by way of a fluorescent reporter assay revealed an average 20% decrease in cleavage of caspases 3 and 7 indicating reduced apoptosis in cells treated with C30- or CP70-derived exosomes as compared to controls (Figure 1D). Finally, a colony formation assay of 200 A2780 cells pre-treated with exosomes for 24 hours prior to exposure to either 1 or 5 μM carboplatin revealed an ~15% increases in the number of colonies when pretreated with exosomes derived from C30 and CP70 cell lines as compared with controls (Figure 1E–1F).

**Figure 2:**
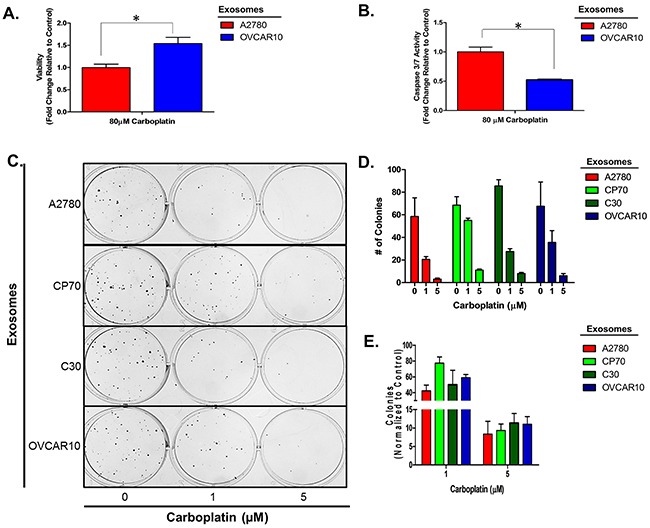
A2780 cell platinum sensitivity is influenced by other platinum-resistant EOC cells **A-B**. Viability (A) and Caspase 3/7 activity (B) cell lines exposed to exosomes derived from autologous or OVCAR10 cell lines and treated with carboplatin for 48 hours. All values are normalized to vehicle. Fold-change is relative to control (autologous treated exo group) **C-F**. Colony formation assay (C) raw data (D) and corresponding analysis (F) of 100 A2780 cells pre-treated for 24 hours with exosomes (autologous, CP70, C30, and OVCAR10) and then exposed to constant levels of 0, 1, or 5 μM carboplatin. All values are normalized to viability. Fold-change is calculated relative to control. *P<0.05.

**Figure 3:**
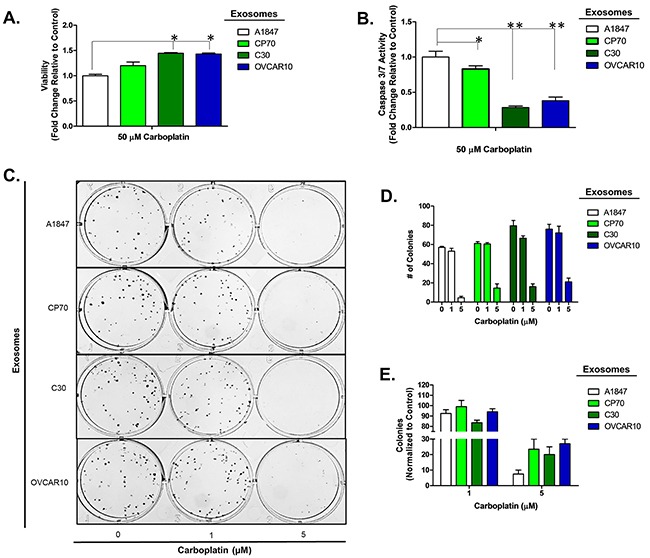
A1847 cell platinum sensitivity is influenced by other platinum-resistant EOC cells **A-B**. Viability (A) and Caspase 3/7 activity (B) cell lines exposed to exosomes derived from autologous, CP70, C30 or OVCAR10 cell lines and treated with carboplatin for 48 hours. All values are normalized to vehicle. Fold-change is relative to control (autologous treated exo group) **C-F**. Colony formation assay (C) raw data (D) and corresponding analysis (F) of 100 A1847 cells pre-treated for 24 hours with exosomes (autologous, CP70, C30, and OVCAR10) and then exposed to constant levels of 0, 1, or 5 μM carboplatin. All values are normalized to viability. Fold-change is calculated relative to control. *P<0.05, **P<0.005.

**Figure 4:**
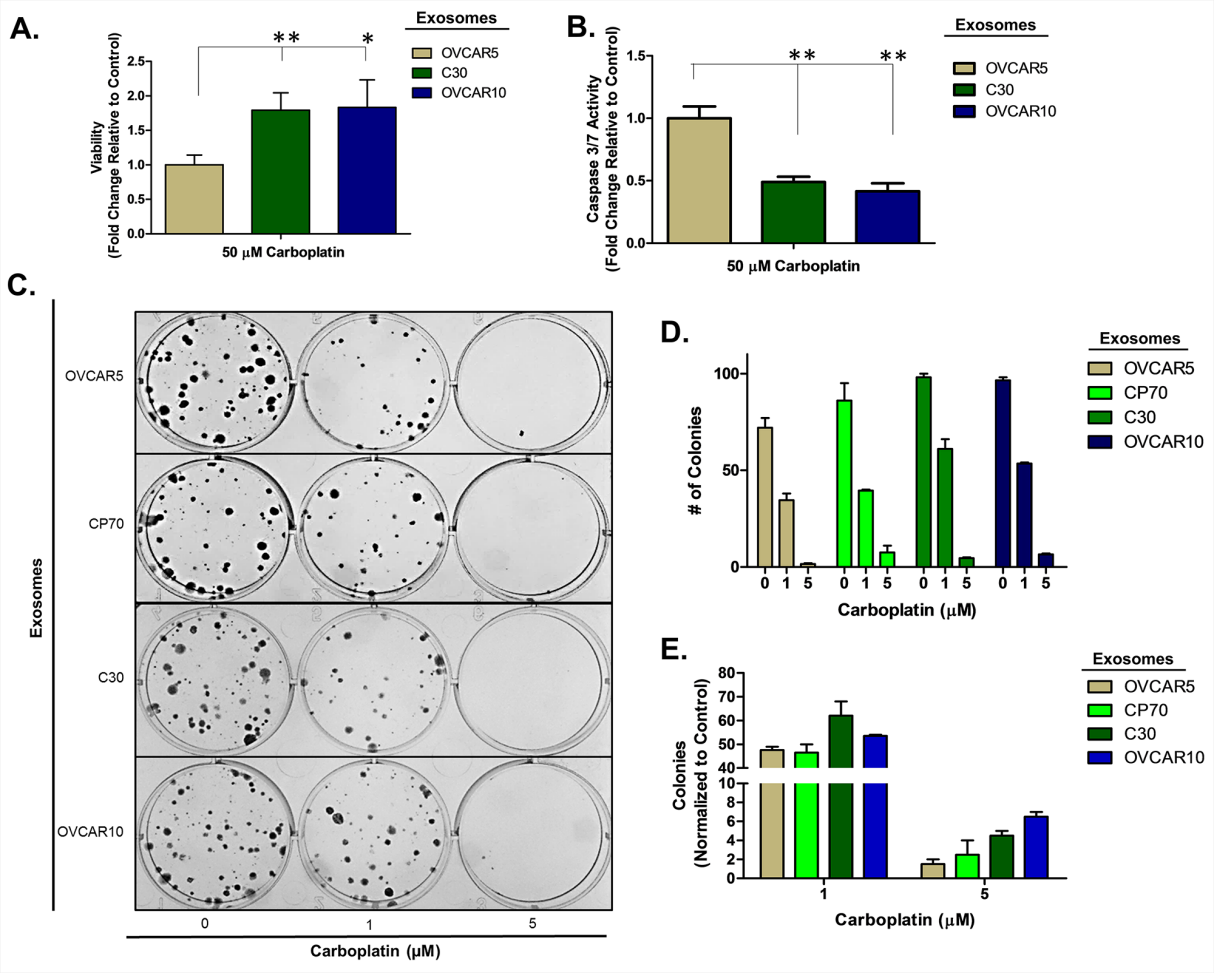
OVCAR5 cell platinum sensitivity is influenced by other platinum-resistant EOC cells **A-B**. Viability (A) and Caspase 3/7 activity (B) cell lines exposed to exosomes derived from autologous, CP70, C30 or OVCAR10 cell lines and treated with carboplatin for 48 hours. All values are normalized to vehicle. Fold-change is relative to control (autologous treated exo group) **C-F**. Colony formation assay (C) raw data (D) and corresponding analysis (F) of 100 OVCAR5 cells pre-treated for 24 hours with exosomes (autologous, CP70, C30, and OVCAR10) and then exposed to constant levels of 0, 1, or 5 μM carboplatin. All values are normalized to viability. Fold-change is calculated relative to control. *P<0.05, **P<0.005.

To confirm this observation is not limited to a select lineage of cells, we investigated the effects of exosomes derived from the unrelated, platinum-resistant OVCAR10 cell line (IC_50_ of 200 μM carboplatin) on A2780 cells and the effects of C30- and OVCAR10-derived exosomes on the A1847 (IC_50_ of 67 μM carboplatin) and OVCAR5 (IC_50_ of 44 μM carboplatin) ovarian cancer cell lines. OVCAR10 was previously derived from a high-dose carboplatin and cisplatin refractory advanced ovarian tumor [11], while A1847 and OVCAR5 were derived from untreated advanced ovarian tumors [41, 46]. Exosomes were isolated and characterized as before (Supplementary Figure 1 and data not shown). Similar to previous observations, A2780 cells treated with OVCAR10-derived exosomes exhibited up to a 1.7-fold (P<0.05) increase in viability as well as a 50% decrease in caspase 3/7 activity following treatment with 80 μM carboplatin for 48 hours (Figure 2A–2B). Likewise, colony formation assays of 100 A2780 cells pre-treated with exosomes for 24 hours demonstrated up to a 20% increase in colony formation upon treatment with 1 μM carboplatin (Figure 2C–2E). We also observed an increase in colony size in A2780 cells treated with exosomes derived from C30 and OVCAR10 upon exposure to 1 μM carboplatin (Supplementary Figure 5). A1847 cells exhibited up to a 1.7-fold (P<0.05) increase in viability and up to a 75% decrease (P<0.005) in caspase 3/7 activity upon exposure to 50 μM carboplatin for 48 hours only in cells treated with exosomes derived from CP70, C30, or OVCAR10 cell lines as compared to autologous exosome controls (Figure 3A–3B). In addition, A1847 cells pre-treated with exosomes derived from CP70, C30, or OVCAR10 derived exosomes, resulted in a near 3-fold increase in colony formation upon exposure to 5 μM carboplatin as compared to controls (Figure 3C–3E). Similar results were seen in OVCAR5 cells, which demonstrated up to a 2-fold increase in viability (P<0.05) and 50% decrease in caspase 3/7 activity (P<0.005) upon exposure to 50 μM carboplatin in cells pre-treated with exosomes derived from C30 and OVCAR10 cell lines as compared to autologous exosome controls (Figure 4A–4B). We also observed up to a 3-fold increase in colony formation in cells treated with exosomes derived from CP70, C30, or OVCAR10 cell lines as compared to autologous exosome controls (Figure 4C–4E). Taken together our data demonstrate the development of platinum resistant disease may, in part, be mediated by cell-cell communication via exosomes.

### Exosomes induce a protracted phenotypic change

**Figure 5:**
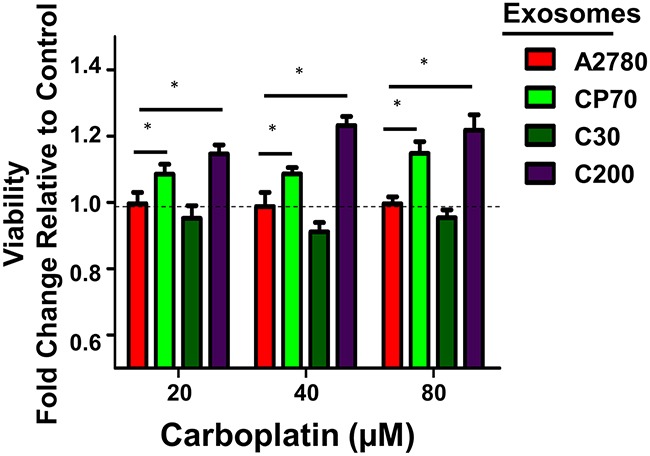
Extended Effects of Exosome Uptake Viability after 72 hours of carboplatin treatment of A2780 cells following a 24 hour exposure to exosomes derived from A2780 (autologous), CP70, C30, or C200 cell lines and normal culture conditions for two weeks. All values are normalized to vehicle. Fold-change is relative to control (A2780-exo group). *P<0.05.

Recent studies by our lab and others have shown the effects of tumor-derived exosomes on recipient neighboring and distal cells can be both transient as well as prolonged and, in some cases, even exert a permanent phenotypic shift [24, 38, 47]. Therefore, we next asked if the acquired platinum-sensitivity profile was durable. We repeated the studies as indicated above and utilized an additional highly platinum-resistant cell line C200 (IC_50_>500 μM), which was derived from C30 by continuous exposure to 200 μM cisplatin [41]. A2780 cells were exposed to autologous, CP70-, C30-, or C200-derived exosomes (1.0 μg exosome/10,000 cells plated), for 24 hours after which the media was replaced and cells were cultured for up to 14 days in standard conditions. Two-weeks after exosome exposure the recipient A2780 cells exposed to C200 or CP70-derived exosomes maintained a more resistant (~1.2-fold increase in viability, P<0.05) phenotype as compared to controls. This increase in resistance was consistent across 20, 40, and 80 μM concentrations of carboplatin (Figure 5).

### Exosomes derived from platinum-resistant cells trigger EMT in platinum-sensitive A2780 cells

**Figure 6:**
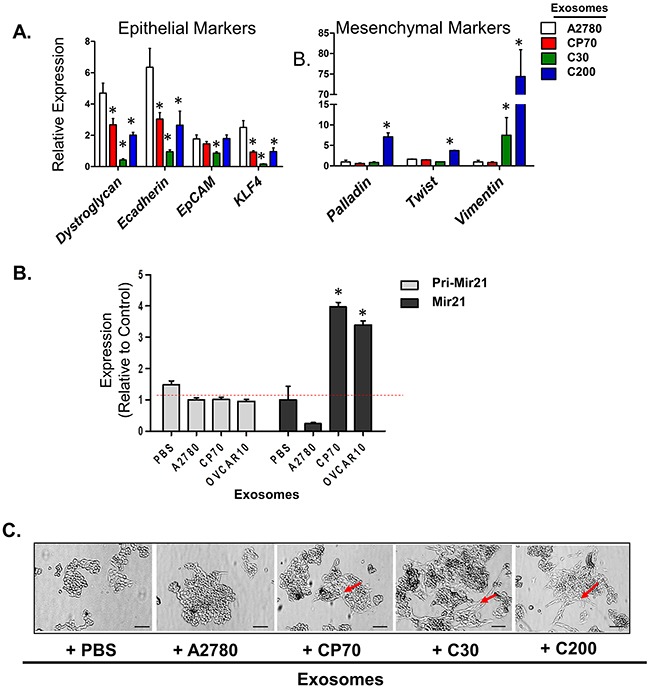
A2780 Cells Undergo EMT When Treated with Exosomes from Platinum-Resistant Cells **A**. Real-time PCR of cDNA from A2780 cells treated with A2780- (autologous), CP70-, C30-, or C200-derived exosomes for 24 hours. All values are given as mRNA levels (*dystroglycan*, *E-cadherin*, *EpCAM*, *KLF4*, *palladin*, *twist*, and *vimentin*) relative to two housekeeping genes (*GAPDH* and *GUSB*). **B**. Real-time PCR of primary miR-21 and mature miR-21 levels in A2780 cells treated with exosomes from A2780 (autologous), CP70, C30, or OVCAR10. Primary miR-21 gene expression is relative to two housekeeping genes (*GAPDH* and *GUSB*). Mature miR-21 expression is relative to U6. **C**. Bright field images of A2780 cells treated with PBS (vehicle) or A2780- (autologous), CP70-, C30-, or C200-derived exosomes detailing enhanced spindle-like and mesenchymal morphology in cells treated with platinum-resistant exosomes as compared to control (arrows). Scale bar = 100 μm. *P<0.05.

Two recent studies have presented evidence which challenges the significance of EMT in cancer metastasis but suggest that inhibition of EMT suppresses chemo-resistance in lung and pancreatic carcinomas [48, 49]. Continuing with this line of thought, there is increasing evidence that EOC cells utilize EMT as a mechanism of escape from the deleterious effects of platinum-based chemotherapy, as evident from the observed up-regulation of mesenchymal markers upon treatment with platinum-based compounds [13, 15, 50]. Likewise, exosomes have been shown to carry cargo which triggers EMT-like changes in breast, colorectal, and urinary cancers [51–53]. Given this information we questioned the effects of exogenous exosome exposure on changes in EMT characteristics. We utilized A2780 and their platinum-resistant derivatives CP70, C30, and C200 cells, which, as we have previously published, all express a basal mesenchymal morphology [14, 54]. A2780 were treated with exosomes as above, and evaluated for changes in expression of mesenchymal markers, including mRNA and miRNA expression as well as changes in morphology. For mRNA analysis we chose a panel of previously published genes to characterize changes in EMT [14]. A2780 cells exposed to exosomes from A2780 platinum-resistant subclones (*i.e*., CP70, C30, or C200) for 24 hours demonstrated at least a 50% reduction (P<0.05) in the expression of the epithelial markers *dystroglycan*, a cell-surface laminin receptor that links the cytoskeleton to the extracellular matrix in a variety of epithelial tissues [55, 56], *E-cadherin*, a cell-cell adhesion molecule and is down-regulated during EMT in numerous cancers [57, 58], and to a lesser extent *EpCAM*, the epithelial cell adhesion molecule expressed in epithelia and epithelial-derived neoplasms [59, 60]. Complimentary to these results we observed significant (P<0.05) increases in the mesenchymal markers *palladin*, which plays a role in modulating the actin cytoskeleton and is ubiquitously expressed in cells of mesenchymal origin [61] and *TWIST*, an embryonic transcription factor which is frequently reactivated in cancer and correlate with poor prognosis. Additionally, we observed up to a 70-fold increase (P<0.05) in *vimentin*, a type III intermediate filament protein expressed in mesenchymal cells [62] in A2780 cells treated with C200-derived exosomes (Figure 6A). We also observed that A2780 cells treated with exosomes from platinum-resistant cells displayed a 2- to 5-fold (P<0.05) decrease in *KLF4*, a transcription factor that regulates genes essential to EMT including *E-cadherin* and *vimentin* and has been shown to regulate EMT in multiple types of cancers [63–66] (Figure 6A).

To further examine markers associated with EMT we examined MicroRNA-21 (miR-21) expression. miR-21 is perhaps, one of the most well studied miRs in regards to EMT in cancer [67–69]. Specifically, in regards to EOC, up-regulation of miR-21 has been shown to correlate with decreased sensitivity to both paclitaxel and cisplatin and has been associated with EOC cell stemness [70–72]. We found that CP70, C30, and OVCAR10 cells have 18-, 5-, and 6-fold increases in mature miR-21 as compared to A2780 cell lines, respectively (Supplementary Figure 6). Importantly, A2780 cells treated with CP70- and OVCAR10-derived exosomes demonstrate a nearly 4-fold increase in miR-21 as compared to cells treated with PBS or autologous-derived exosomes (Figure 6B). Furthermore, there were no significant changes in primary miR-21 (pri-miR-21) levels in A2780 cells treated with exosomes (Figure 6B), suggesting either transfer or increased processing of miR-21 following uptake of exogenous exosomes.

We further observed that, while A2780 cells themselves have a partial mesenchymal phenotype, they displayed an increase in spindle-like morphology when exposed to C30- and C200-derived exosomes, and to a lesser-extent, CP70-derived exosomes (Figure 6C–arrows). Taken together these data demonstrate the transfer of a resistant phenotype may be driven, in part, through transfer or upregulation of factors which contribute to EMT.

### Alterations in SMAD4 are found in platinum-resistant EOC cell lines

**Figure 7:**
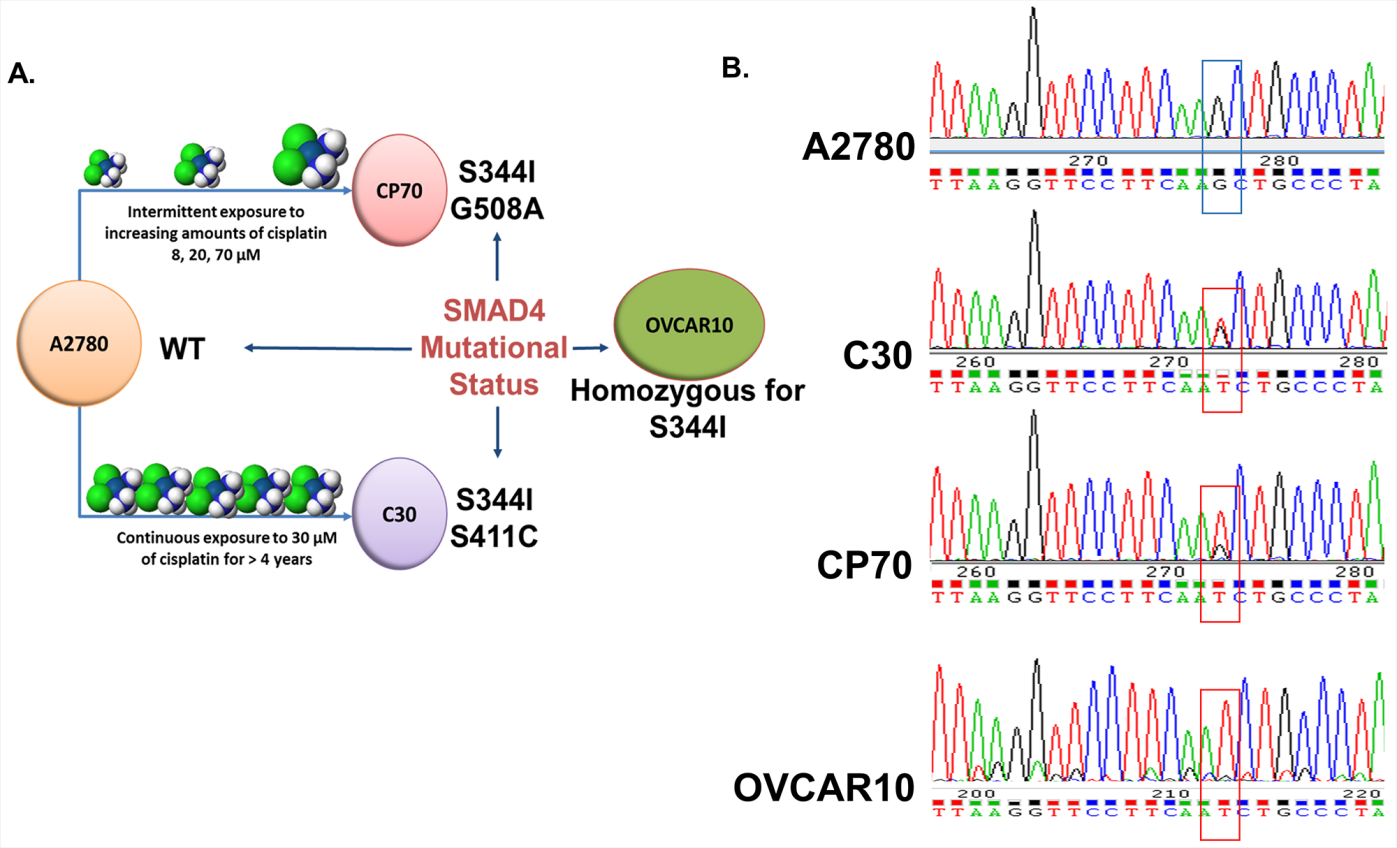
Identification of *SMAD4* mutations in EOC cell lines **A**. Visual schematic of the origin of platinum-resistant CP70, C30 and OVCAR10 cell lines and *SMAD4* mutational status. **B**. Chromatograms of the wild-type *SMAD4* sequence in A2780 cells versus the *SMAD4* S344I (G→T) mutation present in CP70, C30, and OVCAR10 cells.

To identify potential alterations contributing to drug resistance in these models at the genomic level, Next Generation DNA sequencing was performed across 12 ovarian cell lines using a panel of 48 cancer-associated genes using the TruSeq© Amplicon Cancer Panel (Illumina) (Supplementary Table 2). Through this analysis we identified previously unreported somatic mutations in the MH2 domain of *SMAD4*, which were exclusive to the highly platinum resistant cell lines (C30, CP70, and OVCAR10) (Figure 7 and Supplementary Figure 7A). Specifically, all three cell lines, C30, CP70, and OVCAR10, contain a common *SMAD4^S344I^* mutation (OVCAR10 was homozygous for the S344I mutation), while C30 and CP70 each harbor a second unique mutation (*SMAD^S411C^* and *SMAD^G508A^*, respectively) (Figure 7A). The sequencing results were confirmed by sanger sequencing (Figure 7B and data not shown). These initial findings suggest that changes in SMAD4 may contribute or correspond with a loss of platinum sensitivity and warrant further investigation.

We next used *in silico* analysis to determine the potential functional impact of these *SMAD4* mutations [73]. The *SMAD4^Q388R^* variant, a missense change discovered in OVCAR4, a cell line derived from a patient's tumor that was refractory to cisplatin, but with a low level of *in vitro* platinum-resistance [41, 74], received a score of 0.54 and was predicted to have no effect on protein function. The *SMAD4*^S344I^ mutation, which is common to all 3 highly platinum resistant cell lines, was assigned a value of 2.765 and predicted to have a considerable effect on function. The second acquired mutations identified in CP70 (*SMAD4^G508A^*) and C30 (*SMAD4^S411C^*) were both predicted to have a high impact on SMAD4 function (ranked at 3.365 and 3.395, respectively) (Supplementary Figure 7B).

### Dysregulation of TGF-β/SMAD signaling is found in clinical EOC samples

**Figure 8:**
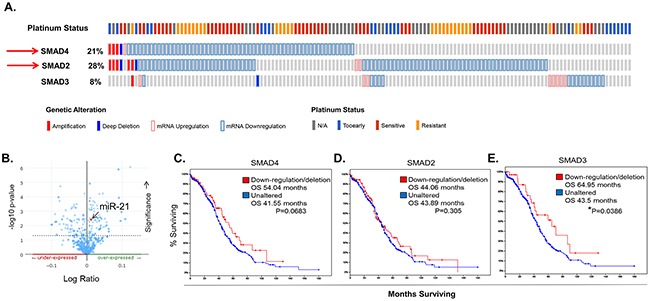
Dysregulation of TGF-β/SMAD Signaling Components in Clinical EOC Samples **A**. Oncoprint generated using the Memorial Sloan Kettering access portal to TCGA EOC data reveals *SMAD2*, *SMAD3*, and *SMAD4* mRNA upregulation (pink outline), mRNA downregulation (lt. blue outline), amplification (solid red), and deletion (solid blue) in 316 EOC samples with complete data (mRNA, copy number alterations (CAN), and sequencing). **B**. miR-21 levels are significantly (p=0.00417) enhanced in TCGA EOC samples with loss of *SMAD4* expression (n=61). Data shown are from 516 EOC cases with microRNA sequencing data. **C-E**. Overall survival plotted as Kaplan-Meier curves that compare patients with down-regulation or deletion of *SMAD2* (C), *SMAD4* (D), or *SMAD3* (E), as compared to unaltered cases. Patients with a loss of *SMAD3* expression have a significant increase (64 months vs 43 months) P=0.0386 in OS and patients with a loss of wild-type *SMAD4* expression have an increased OS of ~12.5 months. P values are determined by log-rank test. P values < 0.05 are considered significant.

SMAD4 is a key component of TGF-β/SMAD signaling. It binds to receptor activated SMADs (SMAD2 or SMAD3), forming a heterodimer which allows for translocation into the nucleus, where it binds additional co-factors and initiates transcription of target genes [75, 76]. To determine how the TGF-β/SMAD signaling pathway might be altered in clinical EOC cases, we used *in silico* data mining approaches and The Cancer Genome Atlas (TCGA) database. We identified multiple genetic aberrations within one or more key components of the TGF-β/SMAD signaling pathway. Most notably, 21% (61 of 316) of high-grade serous EOC samples have deletions and/or down-regulation of *SMAD4* which significantly (p<0.001) correlates with deletions and/or down-regulation of *SMAD2* (deleted or down-regulated in ~28% (81 of 316) of the cases (Figure 8A). In addition, enrichment analysis of samples with available miRNA expression data (n=518) revealed specific miRNAs that are differentially expressed in cases with loss of *SMAD4* as compared to normal controls. These include miRNAs known to impact or be regulated by TGF-β/SMAD signaling, such as miR-142 [77], miR-146A [78, 79], and miR-29B [80]. Importantly, miR-21 was significantly (p=0.00417) overexpressed in cases with a loss of *SMAD4* expression (n=61) (Figure 8B–8arrow). Additionally, out of 316 cases with complete mRNA data, we found no significant change in overall survival (OS) in cases harboring dysregulation in *SMAD4* (n=61) or *SMAD2* (n=81), indicating that loss of *SMAD4* and/or SMAD2 is not a significant predictor of outcome in primary EOC (Figure 8C–8D). However, patients exhibiting a loss of *SMAD3* expression (n=16), albeit small in number, have a significant increase in OS (~44 versus 65 months, p=0.0386), suggesting that activation of the SMAD3 pathway could be important in EOC development (Figure 8E). Continuing with this analysis, *SMAD2* mRNA levels are down-regulated in 32% of TCGA platinum resistant EOC tumors (N=197) as compared to 24% in platinum sensitive (N=90) and *SMAD3* tends to be down-regulated in the platinum sensitive cohort as compared to the resistant (7% and 1% respectively) further highlighting the complexity and potential importance of SMAD signaling in EOC (Supplementary Figure 8). In ovarian cancer, SMAD3 acts alone or in conjunction with SMAD4 to regulate transcription of EMT target genes [81]. Importantly, SMAD4, SMAD3, and other key components of the TGF-β/SMAD signaling pathway (*i.e*., TGFβr1, TGFβr2, and SMAD2) are found within exosomes isolated from tumor cell lines and from human plasma samples (Supplementary Figures 1 & 9).

### Mutant SMAD4 cell lines and exosomes elicit a resistant phenotype in recipient cells

**Figure 9:**
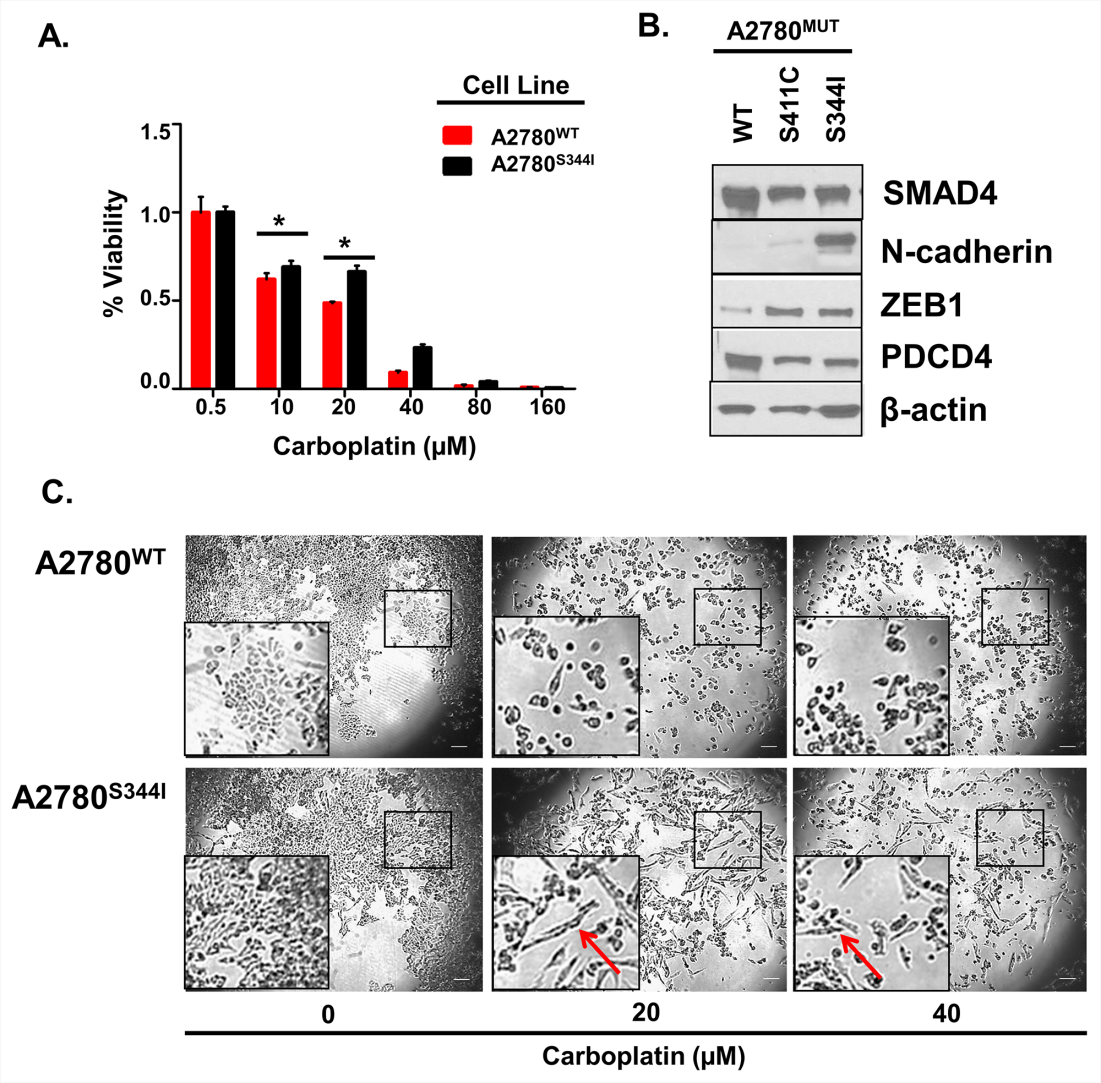
Mutations in *SMAD4* Contribute to Platinum Resistance and EMT **A**. Viability of A2780^WT^ and A2780^S344I^ after exposure to 0, 20, 40, 80, and 160 μM carboplatin for 72 hours. Values are normalized to vehicle (PBS). **B**. Western blot analysis of EMT markers in A2780^WT^, A2780^S411C^, and A2780^S344I^ cell lysates. **C**. Bright field images of A2780^WT^ and A2780^S344I^ after exposure to 0, 20, and 40 μM carboplatin for 24 hours. Arrows indicate changes in morphology. *P<0.05.

To provide direct evidence that mutations in *SMAD4* contribute towards a loss of platinum-sensitivity, we exogenously overexpressed *SMAD4*^S344I^, *SMAD4*^S411C^, or *SMAD4^WT^* in A2780 cell to generate A2780^S344I^, A2780^S411C^, and A2780^WT^ cell lines, respectively. Of these cell lines, A2780^S344I^ demonstrated the highest level of platinum resistance, *e.g*., 2-fold (P<0.05) increase in viability at both 20 and 40 μM carboplatin (Figure 9A). A2780^S344I^ cells also exhibited an increase in mesenchymal markers N-cadherin and ZEB1 as well as a decrease in the mediator of apoptosis, PDCD4 (Programmer of Cell Death 4) (Figure 9B), which is known to regulate EMT [82]. Likewise, we observed a more mesenchymal phenotype upon exposure of A2780^S344I^ cells to 20 and 40 μM carboplatin as compared to A2780^WT^ (Figure 9C), even in the background of the endogenous SMAD4.

**Figure 10:**
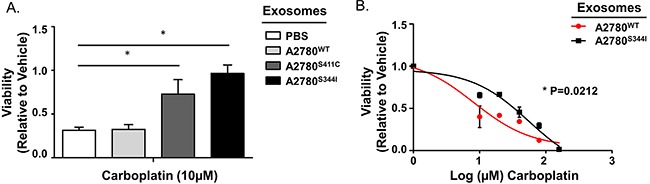
Exosomes from Mutant *SMAD4* Cell Lines Transfer Platinum Resistance **A**. Viability of A2780 cells exposed to PBS or exosomes derived from A2780^WT^, A2780^S411C^, and A2780^S344I^ cell lines for 24 hours prior to carboplatin treatment (10 μM for 72 hours). Viability is normalized to vehicle within each exosome group. **B**. Dose-response curves of A2780 cells pre-exposed to exosomes derived from A2780^WT^ or A2780^S344I^ cell lines for 24 hours followed by carboplatin treatment (0, 10, 20, 40, 80, and 160 μM) for 72 hours. *P<0.05.

To determine if SMAD4^mut^ cell lines produce exosomes capable of transferring a platinum-resistant phenotype, we isolated exosomes from A2780^WT^, A2780^S411C^, and A2780^S344I^ cells as described above. Parental A2780 cells were then exposed to A2780^S344I^-derived, A2780^S411C^-derived, and A2780^WT^-derived exosomes, or PBS. Cells exposed to A2780^S344I^-derived and A2780^S411C^-derived exosomes exhibited near 3-fold and 2-fold increases in viability, respectively, over both controls after exposure to 10 μM carboplatin for 72 hours (Figure 10A). In addition, A2780^S344I^-exosome exposed cells exhibited a nearly 7-fold increase in their IC_50_ values as compared to cells treated with A2780^WT^–exosomes (8.2 μM vs 55.5 μM, respectively) (p=0.0212) (Figure 10B).

Taken together, these data provide the first evidence that mutations, specifically within *SMAD4*, enhance the native chemo-resistance profile of EOC. Specifically, cells harboring these mutations have survival advantages by way of increased EMT in response to carboplatin. In addition, these cells generate and secrete exosomes capable of modifying the platinum-sensitivity profile of surrounding neoplastic cells which enhances the development of platinum-resistant disease.

## DISCUSSION

Because the ovarian tumor mass is composed of a heterogeneous population of cells with a high degree of individual morphologies and genomic instability, the development of platinum-based chemotherapy resistance is multifactorial and frequently due to a variety of changes in specific proteins, genes, or in gene regulation that are advantageous to chemotherapy resistance [10, 83–90]. It is commonly believed that chemotherapeutic treatment used during cancer treatment can cause or select for intrinsically resistant populations of cells which possess one or more of these advantages. For example, Stronah and colleagues have shown that, in response to cisplatin, DNA–dependent protein kinase (DNA-PK) selectively phosphorylates AKT-S473 in the nucleus of platinum-resistant, but not sensitive cells, leading to the clinical development of platinum-resistant disease [91]. Whether by selection of resistant clones or infliction of secondary aberrations, some tumor cells survive primary rounds of therapy, and eventually give rise to new cells which form more therapy resistant tumors. Here we have investigated how drug resistant populations of cells perpetuate the development of platinum-resistant disease in ovarian cancer via exosomal mitigated cell-cell communication. It is well understood that intracellular transport of genetic material, including miRNAs and mRNAs as well as biologically active proteins from one cell to another, occurs via microvesicles [36, 92–96]. We and others have shown that extracellular vesicles may be involved in transformation of surrounding cells via transport of miRNAs and oncogenic proteins [26, 38, 40, 97, 98]. Our current findings suggest that, as the ovarian tumor develops and evolves to escape therapeutic attack, exosomes may be a previously unappreciated factor, which contribute to the advancement of drug resistant disease.

We present evidence demonstrating that exosomes derived from platinum-resistant EOC cells can transfer a portion of their chemo-resistant phenotype to platinum-sensitive cells and this increase in resistance corresponds with increased EMT and mutations in *SMAD4*. SMAD4 has been shown to be necessary for the transcription of EMT related genes through SMAD4/SMAD3 signaling [81] and EMT has been well established as a mediator of drug resistance in ovarian cancer [15, 99–101]. Our focused analysis of TCGA ovarian cancer data did not identify mutations in *SMAD4*; however, we did identify significant dysregulation of key TGF-β/SMAD genes, despite the fact that the vast majority of samples are from primary tumors. The lack of mutations within *SMAD4* is not surprising: however, given that we hypothesize that somatic mutations in *SMAD4* are either selected for or cause by platinum-based chemotherapy. Here we provide the first evidence that mutations within the *SMAD4* gene directly affect the platinum-sensitivity of EOC cells. In addition, we are the first to report that cells harboring these mutations produce exosomes capable of transferring this resistant profile.

TGF-β/SMAD signaling components are found within and on exosomes including SMAD2, SMAD4, TGFβrI and TGFβrII (Supplementary Figures 1 and 9); however, a momentary transfer of protein alone does not provide sufficient evidence to explain a persistent phenotypic switch. Evidence suggests that the transfer of exosomal mRNAs between cells may lead to more prolonged phenotypic changes [102, 103]. We have identified very low levels of the *SMAD4* mRNA within a portion of exosomal samples (Crow and Godwin, unpublished data); however, we were unable to sequence this transcript, suggesting that the whole *SMAD4* mRNA transcript may not be present. Given this information, and the fact that SMAD4 is a transcription factor, we speculate that the exosomal contents responsible for the observed morphological changes may be products of aberrant TGF-β/SMAD signaling.

Current evidence suggests that miR-21 may both be a product of and mediator of TGF-β signaling in many pathological conditions [104–106]. We identified a 3- to 4-fold increase in miR-21 expression in platinum-sensitive cells treated with platinum-resistant exosomes and in the parental platinum-resistant cells. Up-regulation of miR-21 in multiple types of cancers, including ovarian, positively correlates with increased EMT [107–109]. Therefore, a complementary mechanism to alter platinum sensitivity is exosome-induced up-regulation of primary miR-21 (pri-miR-21) in recipient cells. Of relevance, recent studies by Davis and colleagues demonstrate that SMAD proteins can bind with the Drosha complex to increase processing of pri-miR-21 to pre-miR-21 [110]. Indeed, we observed increased miR-21 in A2780 cells treated with platinum-resistant exosomes but no significant changes in pri-miR21, suggesting increased miRNA processing may be an important factor in EOC. Although exciting, further research is warranted to further define the exosomal cargo that is directly responsible for influencing platinum sensitivity.

In summary, we propose a new mechanism by which tumor cell-cell cross talk may actively enhance chemotherapy resistance throughout treatment. It is important to note that the experiments conducted here are limited to a single exosome treatment, and do not mimic the potentially constant exposure to exosomes which likely occurs *in vivo*. This is especially relevant in EOC, where neoplastic cells have enhanced exosomal output as compared to normal epithelial cells [111]. Taken together, this work underscores the importance of cell-cell communication in the advancement of platinum resistant disease and provides novel insight as to how exosomal transfer further enhances EMT in response to frontline chemotherapy.

## MATERIALS AND METHODS

### Cells and culture conditions

We utilized human ovarian cancer cells A2780, C30, CP70, C200, OVCAR5, A1847, and OVCAR10 [11, 43, 112]. Ovarian cancer cell lines A2780, A1847, and OVCAR5 were authenticated by using multiplex short tandem repeat (STR) testing and compared to historical reference DNA preserved in the lab for these cell lines. Testing was performed by the Clinical Molecular Oncology Laboratory at KUMC, a CLIA/CAP-accredited molecular diagnostics laboratory using the Promega PowerPlex 16 System used for human identity testing run on an Applied Biosystems instrument. All cell lines were cultured in RPMI-1640 (Gibco, Thermo Fisher) media supplemented with 10% (v/v) exosome-depleted FBS, 2 mm L-glutamine, 0.2 units/mL human insulin, and 100 units mL penicillin-streptomycin at 37°C with 5% CO_2_. Exosome-depleted FBS was obtained by centrifuging FBS for 18 hours at 100,000 x g followed by filtration through a 0.22 μm filter.

### Exosome isolation

Cell lines were cultured to 70-80% confluency and conditioned media was collected after 24-48 hours, spun for 10 minutes at 2000 x g, and pooled together. Exosomes were isolated by differential centrifugation as previously reported [23]. Briefly media was spun for 45 minutes at 10,000 x g to pellet large vesicles and twice at 100,000x g to pellet and wash exosomes. Exosome pellets were resuspended in 50-100 μL of cold PBS and stored at -80°C.

### Nanoparticle tracking analysis

Purified exosomes were resuspended in 100 μL of 0.22 μm filtered PBS and analyzed using a NanoSight LM10 instrument (NanoSight, Salisbury, United Kingdom). Analysis was performed by applying a monochromatic 404 nm laser to diluted exosomal preparation and measuring the Brownian movements of each particle. The Nanoparticle Tracking Analysis software version 2.3 was used to analyze 60 second videos of data collection to give mean, median, and mode of vesicle size and concentration.

### Electron microscopy

Exosomes were purified as above and fixed using 2% glutaraldehyde in 0.1 M sodium cacodylate buffer at 4°C overnight. The pellet was rinsed in 0.15 M sodium cacodylate buffer (pH 7.4), followed by a post fixation in 1% osmium tetroxide containing 0.1% potassium ferricynide buffered in 0.1 M cacodylate buffer for 1 hour. Exosomes were dehydrated through a series of ethanol washes followed by a propylene oxide bath for 10 minutes. Prepared exosome pellets were embedded in half propylene oxide/half embed 812 resin and cured in a 60°C oven overnight. 80 nm sections were cut using a Leica UC7 ultramicrotome and were picked up on copper thin bar 300-mesh grids and contrasted with 4% uranyl acetate and Sato's lead stain. Samples were examined using a transmission electron microscope JEOL JEM-1400 TEM at 80 KV. Images were captured using a digital camera.

### Exosome uptake and fluorescent microscopy

Exosomes were labeled with PKH67 Green Fluorescent Cell Linker (Sigma Aldrich, St. Louis, MO) in a modified protocol as described previously [38]. A2780 cells were labeled with PKH26 Red Fluorescent Cell Linker (Sigma-Aldrich) according to manufacturer's instructions. Adhered A2780 cells were exposed to exosomes at a concentration of 1 μg/10,000 cells plated in time points of 0.5, 1, 2, and 24 hours. Media was removed upon completion of all time points and cells were gently washed once with PBS and fixed with 4% paraformaldehyde. After washing with PBS, cover slides were attached using a mounting medium containing DAPI. 3-6 images were taken of each time point and overlaid using MetaMorph Software (Molecular Devices, SunnyVale, CA).

### Cell viability assays

Cells were treated with carboplatin (SelleckChem, Houston, TX) diluted from a 20 mM stock in PBS or PBS alone as a vehicle control. Cell viability was assessed using Cell Titer Blue (ThermoFisher), as previously published [113]. Fluorescence was read using the Tecan Plate reader by 560/590 excitation/emission spectra. All assays were conducted in technical triplicates and replicated at least 2 times. Caspase 3/7 activity was conducted using Caspase-Glo^®^ 3/7 (Promega), according to manufacturer's instructions. Statistical significance between experimental and control groups was determined using Student's T-test. Values of < 0.05 were considered significant.

### Colony formation

EOC cells were grown in 10 cm dishes and treated with 10 μg equivalence of exosomes per 100,000 cells for 24 hours. EOC cells were then trypsonized and counted. 100 or 200 cells were plated/well in a 6 well dish and allowed to adhere overnight. The next day either 1 or 5 μM carboplatin was added (PBS was used as a vehicle control). The media + carboplatin was replaced every 7 days. The assay was stopped when a majority of wells grew colonies observable to the naked eye. Media was removed and the cells were washed with PBS prior to fixation with a 3:1 combination of methanol and acetic acid. Colonies were stained with 0.1% crystal violet in methanol. Images of the plates were taken using the AlphaImager™ system and colonies were counted and measured using Adobe™ PhotoShop software.

### SDS page and western blot analysis

Exosome samples and cell lysates (prepared in RIPA buffer) were separated by adding 40 μg protein on 7%, 10%, or 4-20% Mini-PROTEAN^®^ TGX^™^ Precast Gels, (BioRad, Hercules, CA) and transferred to a supported nitrocellulose membrane (BioRad). The membranes were blocked with 5% non-fat milk for one hour. Membranes were incubated with primary antibodies (Supplementary Table 3) overnight and washed thrice for 10 minutes before addition of HRP conjugated anti-rabbit or anti-mouse secondary antibody (BioRad) for 1 hour. Membranes were then washed and treated with ECL Western Blotting Substrate (Fisher Scientific) according to manufacturer's instructions.

### Real time PCR

Cells were harvested at 80% confluency and RNA was extracted using Trizol and Direct-sol™ RNA mini-prep system (Zymo Research, Irvine CA). Reverse transcriptase was performed using the GoScript™ Reverse Transcriptase System (Promega, Madison, WI) according to manufacturer's instructions. Real time PCR was conducted using Sso Advanced™ real-time PCR Master Mix (BioRad). Primers for EMT-related genes were taken from Yew *et al* [14]. For miR-21 identification, TaqMan Probes (Invitrogen) and RT-PCR primers specific to Mir21 (Invitrogen) were used according to manufacturer's instructions. Primers for primary miR-21 were designed as previously published [110].

### Next generation sequencing

### DNA extraction

EOC cells at 80% confluency were trypsonized, collected, and washed 2x with cold PBS. DNA isolation was accomplished using the DNEasy blood and tissue kit (Qiagen, Hilden Germany) according to manufacturer's instructions.

### Library preparation

The TruSeq Amplicon Cancer Panel (Illumina) was used according to manufacturer's instructions. Amplicon libraries were generated by hybridizing pairs of oligonucleotides specific to targeted regions to each DNA sample. Unbound oligonucleotides were removed, and DNA polymerase and ligase were used to connect bound oligonucleotides by extension and ligation. Primers containing index sequences for multiplexing and adapter sequences for cluster generation were used for PCR amplification. Libraries were quantified using the KAPA Library Quantification Kit (KAPA Biosystems, Inc.) specific to Illumina platforms and optimized for the Roche LightCycler 480 ii. Sample libraries were normalized and equal volumes pooled in the final multiplexed sequencing libraries.

### Sequencing and data analysis

Pooled libraries were sequenced on a MiSeq instrument using a 2 × 150 paired-end format using the Custom Amplicon workflow. Base calls were generated on-instrument with the Real Time Analysis (RTA) software (Illumina). Reads were aligned to the Homo Sapiens – UCSC (h19) genome assembly and the Somatic Variant Caller (Illumina) was used for identification of variants. The Illumina Variant Studio software was used to annotate all detected variants. All alternate variant calls were required to have Q scores of at least Q30 and occur at a frequency of ≥ 15%.

### Mutant plasmid generation and transfection

The *SMAD4* plasmid (pcDNA FLAG-SMAD4M; Joan Massague, Addgene) was altered using the Quick Change II Site-Directed Mutagenesis Kit (Agilent, Santa Clara, CA) according to manufacturer's instructions. Primers were designed using the Quick Change Primer Design software (Agilent). All sequence validation was done using Sanger sequencing by GeneWiz, INC. Transfection of plasmids into A2780 cells was accomplished using Lipofectamine® 2000 (Invitrogen) according to manufacturer's instructions. Transfected cells were maintained in media containing 500 μg/mL G418 (Corning).

### Statistical analysis

Statistical analysis was performed using the two tailed Student's t-test on both excel and Graph Pad Prism Programs and one-way ANOVA analysis on Graph Pad Prism.

## SUPPLEMENTARY MATERIALS FIGURES AND TABLES



## Abbreviations

EMT: Epithelial to Mesenchymal Transition
EOC: Epithelial Ovarian Cancer
GIST: Gastrointestinal Stromal Tumors
KLF4: Kruppel Like Factor 4
miR-21: MicroRNA-21
MVB: Multivesicular Body
NTA: Nanoparticle Tracking Analysis
PDCD4: Programmer of Cell Death 4
SMAD2: Mothers Against Decapentaplegic Homolog 2
SMAD3: Mothers Against Decapentaplegic Homolog 3
SMAD4: Mothers Against Decapentaplegic Homolog 4
TCGA: The Cancer Genome Atlas
TGFβrI: Transforming Growth Factor Beta receptor I
TGFβrII: Transforming Growth Factor Beta receptor II

## References

[1] Rossof AH, Talley RW, Stephens R, Thigpen T, Samson MK, Groppe C, Eyre HJ, Fisher R (1979). Phase II evaluation of cis-dichlorodiammineplatinum(II) in advanced malignancies of the genitourinary and gynecologic organs: a Southwest Oncology Group Study. Cancer Treat Rep.

[2] Thigpen T, Shingleton H, Homesley H, LaGasse L, Blessing J (1979). cis-Dichlorodiammineplatinum(II) in the treatment of gynecologic malignancies: phase II trials by the Gynecologic Oncology Group. Cancer Treat Rep.

[3] Alberts DS, Green S, Hannigan EV, OwToole R, Stock-Novack D, Anderson P, Surwit EA, Malvlya VK, Nahhas WA, Jolles CJ (1992). Improved therapeutic index of carboplatin plus cyclophosphamide versus cisplatin plus cyclophosphamide: final report by the Southwest Oncology Group of a phase III randomized trial in stages III and IV ovarian cancer. J Clin Oncol.

[4] Evans BD, Raju KS, Calvert AH, Harland SJ, Wiltshaw E (1983). Phase II study of JM8, a new platinum analog, in advanced ovarian carcinoma. Cancer Treat Rep.

[5] Joss RA, Kaplan S, Goldhirsch A, Sessa C, Brunner KW, Cavalli F (1984). A phase I trial of cis-diammine-1,1-cyclobutane dicarboxylate platinum II (Carboplatin, CBDCA, JM-8) with a single dose every five week-schedule. Invest New Drugs.

[6] McGuire WP, Hoskins WJ, Brady MF, Kucera PR, Partridge EE, Look KY, Clarke-Pearson DL, Davidson M (1996). Cyclophosphamide and cisplatin compared with paclitaxel and cisplatin in patients with stage III and stage IV ovarian cancer. N Engl J Med.

[7] Piccart MJ, Bertelsen K, James K, Cassidy J, Mangioni C, Simonsen E, Stuart G, Kaye S, Vergote I, Blom R, Grimshaw R, Atkinson RJ, Swenerton KD (2000). Randomized intergroup trial of cisplatin-paclitaxel versus cisplatin-cyclophosphamide in women with advanced epithelial ovarian cancer: three-year results. J Natl Cancer Inst.

[8] Kyrgiou M, Salanti G, Pavlidis N, Paraskevaidis E, Ioannidis JP (2006). Survival benefits with diverse chemotherapy regimens for ovarian cancer: meta-analysis of multiple treatments. J Natl Cancer Inst.

[9] Galluzzi L, Senovilla L, Vitale I, Michels J, Martins I, Kepp O, Castedo M, Kroemer G (2012). Molecular mechanisms of cisplatin resistance. Oncogene.

[10] Stewart DJ (2007). Mechanisms of resistance to cisplatin and carboplatin. Crit Rev Oncol Hematol.

[11] Hamilton TC, Lai GM, Rothenberg ML, Fojo AT, Young RC, Ozols RF (1989). Mechanisms of resistance to cisplatin and alkylating agents. Cancer Treat Res.

[12] Perez RP, Hamilton TC, Ozols RF (1990). Resistance to alkylating agents and cisplatin: insights from ovarian carcinoma model systems. Pharmacol Ther.

[13] Marchini S, Fruscio R, Clivio L, Beltrame L, Porcu L, Nerini IF, Cavalieri D, Chiorino G, Cattoretti G, Mangioni C, Milani R, Torri V, Romualdi C (2013). Resistance to platinum-based chemotherapy is associated with epithelial to mesenchymal transition in epithelial ovarian cancer. Eur J Cancer.

[14] Yew KH, Crow J, Hirst J, Pressetto Z, Godwin AK (2013). Epimorphin-induced MET sensitizes ovarian cancer cells to platinum. PLoS One.

[15] Latifi A, Abubaker K, Castrechini N, Ward AC, Liongue C, Dobill F, Kumar J, Thompson EW, Quinn MA, Findlay JK, Ahmed N (2011). Cisplatin treatment of primary and metastatic epithelial ovarian carcinomas generates residual cells with mesenchymal stem cell-like profile. J Cell Biochem.

[16] Wang Y, Niu XL, Guo XQ, Yang J, Li L, Qu Y, C Xiu Hu, Mao LQ, Wang D (2015). IL6 induces TAM resistance via kinase-specific phosphorylation of ERalpha in OVCA cells. J Mol Endocrinol.

[17] Coffman LG, Choi YJ, McLean K, Allen BL, di Magliano MP, Buckanovich RJ (2016). Human carcinoma-associated mesenchymal stem cells promote ovarian cancer chemotherapy resistance via a BMP4/HH signaling loop. Oncotarget.

[18] Sung PL, Jan YH, Lin SC, Huang CC, Lin H, Wen KC, Chao KC, Lai CR, Wang PH, Chuang CM, Wu HH, Twu NF, Yen MS (2016). Periostin in tumor microenvironment is associated with poor prognosis and platinum resistance in epithelial ovarian carcinoma. Oncotarget.

[19] Dijkgraaf EM, Heusinkveld M, Tummers B, Vogelpoel LT, Goedemans R, Jha V, Nortier JW, Welters MJ, Kroep JR, van der Burg SH (2013). Chemotherapy alters monocyte differentiation to favor generation of cancer-supporting M2 macrophages in the tumor microenvironment. Cancer Res.

[20] Kwon Y, Smith BD, Zhou Y, Kaufman MD, Godwin AK (2015). Effective inhibition of c-MET-mediated signaling, growth and migration of ovarian cancer cells is influenced by the ovarian tissue microenvironment. Oncogene.

[21] Keller S, Konig AK, Marme F, Runz S, Wolterink S, Koensgen D, Mustea A, Sehouli J, Altevogt P (2009). Systemic presence and tumor-growth promoting effect of ovarian carcinoma released exosomes. Cancer Lett.

[22] Stoeck A, Keller S, Riedle S, Sanderson MP, Runz S, Le Naour F, Gutwein P, Ludwig A, Rubinstein E, Altevogt P (2006). A role for exosomes in the constitutive and stimulus-induced ectodomain cleavage of L1 and CD44. Biochem J.

[23] Thery C, Zitvogel L, Amigorena S (2002). Exosomes: composition, biogenesis and function. Nat Rev Immunol.

[24] ZY Abd Elmageed, Yang Y, Thomas R, Ranjan M, Mondal D, Moroz K, Fang Z, Rezk BM, Moparty K, Sikka SC, Sartor O, Abdel-Mageed AB (2014). Neoplastic reprogramming of patient-derived adipose stem cells by prostate cancer cell-associated exosomes. Stem Cells.

[25] Schneider A, Simons M (2012). Exosomes: vesicular carriers for intercellular communication in neurodegenerative disorders. Cell Tissue Res.

[26] Roberson CD, Atay S, Gercel-Taylor C, Taylor DD (2010). Tumor-derived exosomes as mediators of disease and potential diagnostic biomarkers. Cancer Biomark.

[27] Pan BT, Teng K, Wu C, Adam M, Johnstone RM (1985). Electron microscopic evidence for externalization of the transferrin receptor in vesicular form in sheep reticulocytes. J Cell Biol.

[28] Henderson MC, Azorsa DO (2012). The genomic and proteomic content of cancer cell-derived exosomes. Front Oncol.

[29] Lasser C, Eldh M, Lotvall J (2012). Isolation and characterization of RNA-containing exosomes. J Vis Exp.

[30] Crow J, Samuel G, Atay S, Godwin AK (2016). Exosomes: Mediators of Cell-Cell Communication and Potential Biomarkers in Cancer.

[31] Vlassov AV, Magdaleno S, Setterquist R, Conrad R (2012). Exosomes: Current knowledge of their composition, biological functions, and diagnostic and therapeutic potentials. Biochim Biophys Acta.

[32] Cho JA, Park H, Lim EH, Kim KH, Choi JS, Lee JH, Shin JW, Lee KW (2011). Exosomes from ovarian cancer cells induce adipose tissue-derived mesenchymal stem cells to acquire the physical and functional characteristics of tumor-supporting myofibroblasts. Gynecol Oncol.

[33] Peng P, Yan Y, Keng S (2011). Exosomes in the ascites of ovarian cancer patients: origin and effects on anti-tumor immunity. Oncol Rep.

[34] Corcoran C, Rani S, O’Brien K, O’Neill A, Prencipe M, Sheikh R, Webb G, McDermott R, Watson W, Crown J, O’Driscoll L (2012). Docetaxel-resistance in prostate cancer: evaluating associated phenotypic changes and potential for resistance transfer via exosomes. PLoS One.

[35] Xiao X, Yu S, Li S, Wu J, Ma R, Cao H, Zhu Y, Feng J (2014). Exosomes: decreased sensitivity of lung cancer A549 cells to cisplatin. PLoS One.

[36] Chen WX, Liu XM, Lv MM, Chen L, Zhao JH, Zhong SL, Ji MH, Hu Q, Luo Z, Wu JZ, Tang JH (2014). Exosomes from Drug-Resistant Breast Cancer Cells Transmit Chemoresistance by a Horizontal Transfer of MicroRNAs. PLoS One.

[37] Peinado H, Aleckovic M, Lavotshkin S, Matei I, Costa-Silva B, Moreno-Bueno G, Hergueta-Redondo M, Williams C, Garcia-Santos G, Ghajar CM, Nitadori-Hoshino A, Hoffman C, Badal K (2012). Melanoma exosomes educate bone marrow progenitor cells toward a pro-metastatic phenotype through MET. Nat Med.

[38] Atay S, Banskota S, Crow J, Sethi G, Rink L, Godwin AK (2014). Oncogenic KIT-containing exosomes increase gastrointestinal stromal tumor cell invasion. Proc Natl Acad Sci U S A.

[39] Gorczynski RM, Erin N, Zhu F (2016). Serum-derived exosomes from mice with highly metastatic breast cancer transfer increased metastatic capacity to a poorly metastatic tumor. Cancer Med.

[40] Rodriguez M, Silva J, Herrera A, Herrera M, Pena C, Martin P, Gil-Calderon B, Larriba MJ, Coronado MJ, Soldevilla B, Turrion VS, Provencio M, Sanchez A (2015). Exosomes enriched in stemness/metastatic-related mRNAS promote oncogenic potential in breast cancer. Oncotarget.

[41] Godwin AK, Meister A, O’Dwyer PJ, Huang CS, Hamilton TC, Anderson ME (1992). High resistance to cisplatin in human ovarian cancer cell lines is associated with marked increase of glutathione synthesis. Proc Natl Acad Sci U S A.

[42] Louie KG, Behrens BC, Kinsella TJ, Hamilton TC, Grotzinger KR, McKoy WM, Winker MA, Ozols RF (1985). Radiation survival parameters of antineoplastic drug-sensitive and -resistant human ovarian cancer cell lines and their modification by buthionine sulfoximine. Cancer Res.

[43] Behrens BC, Hamilton TC, Masuda H, Grotzinger KR, Whang-Peng J, Louie KG, Knutsen T, McKoy WM, Young RC, Ozols RF (1987). Characterization of a cis-diamminedichloroplatinum(II)-resistant human ovarian cancer cell line and its use in evaluation of platinum analogues. Cancer Res.

[44] Johnstone RM, Bianchini A, Teng K (1989). Reticulocyte maturation and exosome release: transferrin receptor containing exosomes shows multiple plasma membrane functions. Blood.

[45] Raposo G, Tenza D, Mecheri S, Peronet R, Bonnerot C, Desaymard C (1997). Accumulation of major histocompatibility complex class II molecules in mast cell secretory granules and their release upon degranulation. Mol Biol Cell.

[46] Eva A, Robbins KC, Andersen PR, Srinivasan A, Tronick SR, Reddy EP, Ellmore NW, Galen AT, Lautenberger JA, Papas TS, Westin EH, Wong-Staal F, Gallo RC (1982). Cellular genes analogous to retroviral onc genes are transcribed in human tumour cells. Nature.

[47] Ogorevc E, Kralj-Iglic V, Veranic P (2013). The role of extracellular vesicles in phenotypic cancer transformation. Radiol Oncol.

[48] Fischer KR, Durrans A, Lee S, Sheng J, Li F, Wong ST, Choi H, El Rayes T, Ryu S, Troeger J, Schwabe RF, Vahdat LT, Altorki NK (2015). Epithelial-to-mesenchymal transition is not required for lung metastasis but contributes to chemoresistance. Nature.

[49] Zheng X, Carstens JL, Kim J, Scheible M, Kaye J, Sugimoto H, Wu CC, LeBleu VS, Kalluri R (2015). Epithelial-to-mesenchymal transition is dispensable for metastasis but induces chemoresistance in pancreatic cancer. Nature.

[50] Kurokawa M, Ise N, Omi K, Goishi K, Higashiyama S (2013). Cisplatin influences acquisition of resistance to molecular-targeted agents through epithelial-mesenchymal transition-like changes. Cancer Sci.

[51] Franzen CA, Blackwell RH, Todorovic V, Greco KA, Foreman KE, Flanigan RC, Kuo PC, Gupta GN (2015). Urothelial cells undergo epithelial-to-mesenchymal transition after exposure to muscle invasive bladder cancer exosomes. Oncogenesis.

[52] Galindo-Hernandez O, Serna-Marquez N, Castillo-Sanchez R, Salazar EP (2014). Extracellular vesicles from MDA-MB-231 breast cancer cells stimulated with linoleic acid promote an EMT-like process in MCF10A cells. Prostaglandins Leukot Essent Fatty Acids.

[53] Philip R, Heiler S, Mu W, Buchler MW, Zoller M, Thuma F (2015). Claudin-7 promotes the epithelial-mesenchymal transition in human colorectal cancer. Oncotarget.

[54] Kwon Y, Cukierman E, Godwin AK (2011). Differential expressions of adhesive molecules and proteases define mechanisms of ovarian tumor cell matrix penetration/invasion. PLoS One.

[55] Sgambato A, Migaldi M, Montanari M, Camerini A, Brancaccio A, Rossi G, Cangiano R, Losasso C, Capelli G, Trentini GP, Cittadini A (2003). Dystroglycan expression is frequently reduced in human breast and colon cancers and is associated with tumor progression. Am J Pathol.

[56] Mathew G, Mitchell A, Down JM, Jacobs LA, Hamdy FC, Eaton C, Rosario DJ, Cross SS, Winder SJ (2013). Nuclear targeting of dystroglycan promotes the expression of androgen regulated transcription factors in prostate cancer. Sci Rep.

[57] Kalluri R, Weinberg RA (2009). The basics of epithelial-mesenchymal transition. J Clin Invest.

[58] Lamouille S, Xu J, Derynck R (2014). Molecular mechanisms of epithelial-mesenchymal transition. Nat Rev Mol Cell Biol.

[59] Hyun KA, Koo GB, Han H, Sohn J, Choi W, Kim SI, Jung HI, Kim YS (2016). Epithelial-to-mesenchymal transition leads to loss of EpCAM and different physical properties in circulating tumor cells from metastatic breast cancer. Oncotarget.

[60] van der Gun BT, Melchers LJ, Ruiters MH, de Leij LF, McLaughlin PM, Rots MG (2010). EpCAM in carcinogenesis: the good, the bad or the ugly. Carcinogenesis.

[61] Otey CA, Rachlin A, Moza M, Arneman D, Carpen O (2005). The palladin/myotilin/myopalladin family of actin-associated scaffolds. Int Rev Cytol.

[62] Clarke EJ, Allan V (2002). Intermediate filaments: vimentin moves in. Curr Biol.

[63] Cui J, Shi M, Quan M, Xie K (2013). Regulation of EMT by KLF4 in gastrointestinal cancer. Curr Cancer Drug Targets.

[64] Li H, Wang J, Xiao W, Xia D, Lang B, Wang T, Guo X, Hu Z, Ye Z, Xu H (2014). Epigenetic inactivation of KLF4 is associated with urothelial cancer progression and early recurrence. J Urol.

[65] Tiwari N, Meyer-Schaller N, Arnold P, Antoniadis H, Pachkov M, van Nimwegen E, Christofori G (2013). Klf4 is a transcriptional regulator of genes critical for EMT, including Jnk1 (Mapk8). PLoS One.

[66] Chen Z, Wang Y, Liu W, Zhao G, Lee S, Balogh A, Zou Y, Guo Y, Zhang Z, Gu W, Li C, Tigyi G, Yue J (2014). Doxycycline inducible Kruppel-like factor 4 lentiviral vector mediates mesenchymal to epithelial transition in ovarian cancer cells. PLoS One.

[67] De Mattos-Arruda L, Bottai G, Nuciforo PG, Di Tommaso L, Giovannetti E, Peg V, Losurdo A, Perez-Garcia J, Masci G, Corsi F, Cortes J, Seoane J, Calin GA (2015). MicroRNA-21 links epithelial-to-mesenchymal transition and inflammatory signals to confer resistance to neoadjuvant trastuzumab and chemotherapy in HER2-positive breast cancer patients. Oncotarget.

[68] Luo F, Ji J, Liu Y, Xu Y, Zheng G, Jing J, Wang B, Xu W, Shi L, Lu X, Liu Q (2014). MicroRNA-21, up-regulated by arsenite, directs the epithelial-mesenchymal transition and enhances the invasive potential of transformed human bronchial epithelial cells by targeting PDCD4. Toxicol Lett.

[69] Cao J, Liu J, Xu R, Zhu X, Liu L, Zhao X (2016). MicroRNA-21 stimulates epithelial-to-mesenchymal transition and tumorigenesis in clear cell renal cells. Mol Med Rep.

[70] Chan JK, Blansit K, Kiet T, Sherman A, Wong G, Earle C, Bourguignon LY (2014). The inhibition of miR-21 promotes apoptosis and chemosensitivity in ovarian cancer. Gynecol Oncol.

[71] Echevarria-Vargas IM, Valiyeva F, Vivas-Mejia PE (2014). Upregulation of miR-21 in cisplatin resistant ovarian cancer via JNK-1/c-Jun pathway. PLoS One.

[72] Kang HY (2013). MicroRNA-21 regulates stemness in cancer cells. Stem Cell Res Ther.

[73] Reva B, Antipin Y, Sander C (2011). Predicting the functional impact of protein mutations: application to cancer genomics. Nucleic Acids Res.

[74] Godwin AK, Testa JR, Handel LM, Liu Z, Vanderveer LA, Tracey PA, Hamilton TC (1992). Spontaneous transformation of rat ovarian surface epithelial cells: association with cytogenetic changes and implications of repeated ovulation in the etiology of ovarian cancer. J Natl Cancer Inst.

[75] Padua D, Massague J (2009). Roles of TGFbeta in metastasis. Cell Res.

[76] Massague J (2008). TGFbeta in Cancer. Cell.

[77] Lei Z, Xu G, Wang L, Yang H, Liu X, Zhao J, Zhang HT (2014). MiR-142-3p represses TGF-beta-induced growth inhibition through repression of TGFbetaR1 in non-small cell lung cancer. FASEB J.

[78] Santos JI, Teixeira AL, Dias F, Gomes M, Nogueira A, Assis J, Medeiros R (2014). Restoring TGFbeta1 pathway-related microRNAs: possible impact in metastatic prostate cancer development. Tumour Biol.

[79] Xiao B, Zhu ED, Li N, Lu DS, Li W, Li BS, Zhao YL, Mao XH, Guo G, Yu PW, Zou QM (2012). Increased miR-146a in gastric cancer directly targets SMAD4 and is involved in modulating cell proliferation and apoptosis. Oncol Rep.

[80] Yan MD, Yao CJ, Chow JM, Chang CL, Hwang PA, Chuang SE, Whang-Peng J, Lai GM (2015). Fucoidan Elevates MicroRNA-29b to Regulate DNMT3B-MTSS1 Axis and Inhibit EMT in Human Hepatocellular Carcinoma Cells. Mar Drugs.

[81] Do TV, Kubba LA, Du H, Sturgis CD, Woodruff TK (2008). Transforming growth factor-beta1, transforming growth factor-beta2, and transforming growth factor-beta3 enhance ovarian cancer metastatic potential by inducing a Smad3-dependent epithelial-to-mesenchymal transition. Mol Cancer Res.

[82] Wang Q, Zhu J, Zhang Y, Sun Z, Guo X, Wang X, Lee E, Bakthavatchalu V, Yang Q, Yang HS (2013). Down-regulation of programmed cell death 4 leads to epithelial to mesenchymal transition and promotes metastasis in mice. Eur J Cancer.

[83] GTaI Diaz-Padilla, Diaz-Padilla DI (2013). Molecular Mechanisms of Platinum Resistance in Ovarian Cancer, Ovarian Cancer- A Clinical and Translational Update.

[84] Barr MP, Gray SG, Hoffmann AC, Hilger RA, Thomale J, O’Flaherty JD, Fennell DA, Richard D, O’Leary JJ, O’Byrne KJ (2013). Generation and characterisation of cisplatin-resistant non-small cell lung cancer cell lines displaying a stem-like signature. PLoS One.

[85] Mir R, Tortosa A, Martinez-Soler F, Vidal A, Condom E, Perez-Perarnau A, Ruiz-Larroya T, Gil J, Gimenez-Bonafe P (2012). Mdm2 antagonists induce apoptosis and synergize with cisplatin overcoming chemoresistance in TP53 wild type ovarian cancer cells. Int J Cancer.

[86] Shang X, Lin X, Manorek G, Howell SB (2012). Claudin-3 and Claudin-4 Regulate Sensitivity to Cisplatin by Controlling Expression of the Copper and Cisplatin Influx Rransporter CTR1. Mol Pharmacol.

[87] Guddati AK (2012). Ovarian cancer stem cells: elusive targets for chemotherapy. Med Oncol.

[88] Cohen S, Bruchim I, Graiver D, Evron Z, Oron-Karni V, Pasmanik-Chor M, Eitan R, Bernheim J, Levavi H, Fishman A, Flescher E (2012). Platinum-resistance in ovarian cancer cells is mediated by IL-6 secretion via the increased expression of its target cIAP-2. J Mol Med (Berl).

[89] Eckstein N, Servan K, Hildebrandt B, Politz A, von Jonquieres G, Wolf-Kummeth S, Napierski I, Hamacher A, Kassack MU, Budczies J, Beier M, Dietel M, Royer-Pokora B (2009). Hyperactivation of the insulin-like growth factor receptor I signaling pathway is an essential event for cisplatin resistance of ovarian cancer cells. Cancer Res.

[90] Eitan R, Kushnir M, Lithwick-Yanai G, David MB, Hoshen M, Glezerman M, Hod M, Sabah G, Rosenwald S, Levavi H (2009). Tumor microRNA expression patterns associated with resistance to platinum based chemotherapy and survival in ovarian cancer patients. Gynecol Oncol.

[91] Stronach EA, Chen M, Maginn EN, Agarwal R, Mills GB, Wasan H, Gabra H (2011). DNA-PK mediates AKT activation and apoptosis inhibition in clinically acquired platinum resistance. Neoplasia.

[92] Boelens MC, Wu TJ, Nabet BY, Xu B, Qiu Y, Yoon T, Azzam DJ, C Twyman-Saint Victor, Wiemann BZ, Ishwaran H, PJ Ter Brugge, Jonkers J, Slingerland J (2014). Exosome transfer from stromal to breast cancer cells regulates therapy resistance pathways. Cell.

[93] Chen WX, Cai YQ, Lv MM, Chen L, Zhong SL, Ma TF, Zhao JH, Tang JH (2014). Exosomes from docetaxel-resistant breast cancer cells alter chemosensitivity by delivering microRNAs. Tumour Biol.

[94] Federici C, Petrucci F, Caimi S, Cesolini A, Logozzi M, Borghi M, D’Ilio S, Lugini L, Violante N, Azzarito T, Majorani C, Brambilla D, Fais S (2014). Exosome release and low pH belong to a framework of resistance of human melanoma cells to cisplatin. PLoS One.

[95] Lv MM, Zhu XY, Chen WX, Zhong SL, Hu Q, Ma TF, Zhang J, Chen L, Tang JH, Zhao JH (2014). Exosomes mediate drug resistance transfer in MCF-7 breast cancer cells and a probable mechanism is delivery of P-glycoprotein. Tumour Biol.

[96] Takahashi K, Yan IK, Kogure T, Haga H, Patel T (2014). Extracellular vesicle-mediated transfer of long non-coding RNA ROR modulates chemosensitivity in human hepatocellular cancer. FEBS Open Bio.

[97] Maida Y, Takakura M, Nishiuchi T, Yoshimoto T, Kyo S (2016). Exosomal transfer of functional small RNAs mediates cancer-stroma communication in human endometrium. Cancer Med.

[98] Zhang L, Zhang S, Yao J, Lowery FJ, Zhang Q, Huang WC, Li P, Li M, Wang X, Zhang C, Wang H, Ellis K, Cheerathodi M (2015). Microenvironment-induced PTEN loss by exosomal microRNA primes brain metastasis outgrowth. Nature.

[99] Smolle E, Taucher V, Petru E, Haybaeck J (2014). Targeted treatment of ovarian cancer--the multiple - kinase - inhibitor sorafenib as a potential option. Anticancer Res.

[100] Helleman J, Smid M, Jansen MP, van der Burg ME, Berns EM (2010). Pathway analysis of gene lists associated with platinum-based chemotherapy resistance in ovarian cancer: the big picture. Gynecol Oncol.

[101] Marchini S, Fruscio R, Clivio L, Beltrame L, Porcu L, I Fuso Nerini, Cavalieri D, Chiorino G, Cattoretti G, Mangioni C, Milani R, Torri V, Romualdi C (2013). Resistance to platinum-based chemotherapy is associated with epithelial to mesenchymal transition in epithelial ovarian cancer. Eur J Cancer.

[102] Deregibus MC, Cantaluppi V, Calogero R, M Lo Iacono, Tetta C, Biancone L, Bruno S, Bussolati B, Camussi G (2007). Endothelial progenitor cell derived microvesicles activate an angiogenic program in endothelial cells by a horizontal transfer of mRNA. Blood.

[103] Valadi H, Ekstrom K, Bossios A, Sjostrand M, Lee JJ, Lotvall JO (2007). Exosome-mediated transfer of mRNAs and microRNAs is a novel mechanism of genetic exchange between cells. Nat Cell Biol.

[104] Han M, Wang F, Gu Y, Pei X, Guo G, Yu C, Li L, Zhu M, Xiong Y, Wang Y (2016). MicroRNA-21 induces breast cancer cell invasion and migration by suppressing smad7 via EGF and TGF-beta pathways. Oncol Rep.

[105] Lai JY, Luo J, O’Connor C, Jing X, Nair V, Ju W, Randolph A, Ben-Dov IZ, Matar RN, Briskin D, Zavadil J, Nelson RG, Tuschl T (2015). MicroRNA-21 in glomerular injury. J Am Soc Nephrol.

[106] Garcia R, Nistal JF, Merino D, Price NL, Fernandez-Hernando C, Beaumont J, Gonzalez A, Hurle MA, Villar AV (2015). p-SMAD2/3 and DICER promote pre-miR-21 processing during pressure overload-associated myocardial remodeling. Biochim Biophys Acta.

[107] Wang JY, Gao YB, Zhang N, Zou DW, Wang P, Zhu ZY, Li JY, Zhou SN, Wang SC, Wang YY, Yang JK (2014). miR-21 overexpression enhances TGF-beta1-induced epithelial-to-mesenchymal transition by target smad7 and aggravates renal damage in diabetic nephropathy. Mol Cell Endocrinol.

[108] Zhang Y, Pan T, Zhong X, Cheng C (2014). Nicotine upregulates microRNA-21 and promotes TGF-beta-dependent epithelial-mesenchymal transition of esophageal cancer cells. Tumour Biol.

[109] Zhao J, Tang N, Wu K, Dai W, Ye C, Shi J, Zhang J, Ning B, Zeng X, Lin Y (2014). MiR-21 Simultaneously Regulates ERK1 Signaling in HSC Activation and Hepatocyte EMT in Hepatic Fibrosis. PLoS One.

[110] Davis BN, Hilyard AC, Lagna G, Hata A (2008). SMAD proteins control DROSHA-mediated microRNA maturation. Nature.

[111] Gercel-Taylor C, Atay S, Tullis RH, Kesimer M, Taylor DD (2012). Nanoparticle analysis of circulating cell-derived vesicles in ovarian cancer patients. Anal Biochem.

[112] Perez RP, Perez KM, Handel LM, In Hamilton TC (1992). vitro interactions between platinum analogues in human ovarian-carcinoma cell lines. Cancer Chemother Pharmacol.

[113] Sethi G, Pathak HB, Zhang H, Zhou Y, Einarson MB, Vathipadiekal V, Gunewardena S, Birrer MJ, Godwin AK (2012). An RNA interference lethality screen of the human druggable genome to identify molecular vulnerabilities in epithelial ovarian cancer. PLoS One.

