# Supplementary material for: Exosomes as mediators of platinum resistance in ovarian cancer
Source: Oncotarget. 2017 Jan 2;8(7):11917–36. doi: 10.18632/oncotarget.14440 (PMC5355315; doi:10.18632/oncotarget.14440)
Supplement: Supplementary file 1 [file oncotarget-08-11917-s001.pdf]

Exosomes as mediators of platinum resistance in ovarian cancer

SUPPLEMENTARY FIGURES AND TABLES

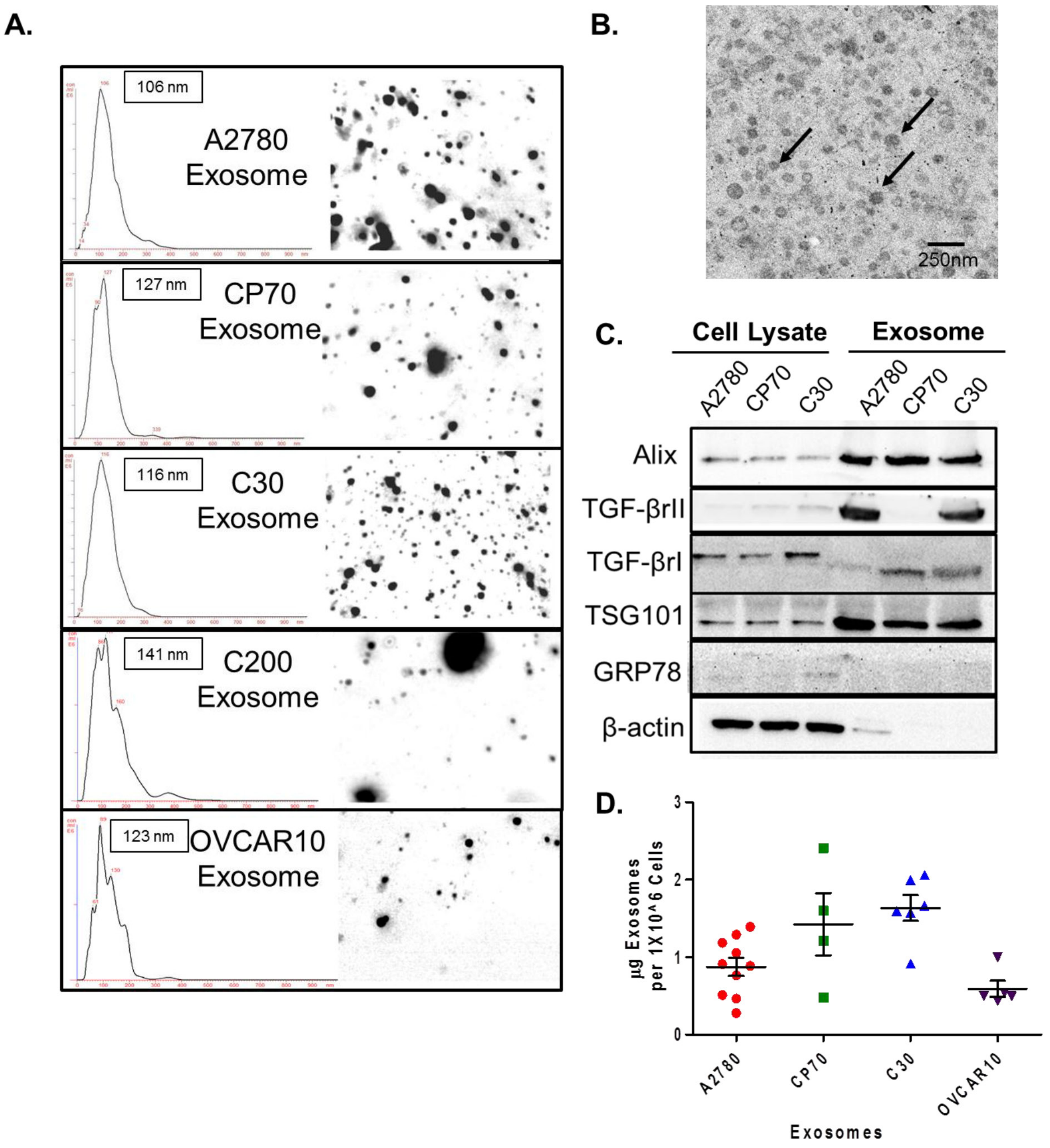

Supplementary Figure 1: Characterization of EOC Derived Exosomes. A. Nanoparticle analysis of exosomes derived from EOC cell lines A2780, CP70, C30, C200, and OVCAR10 shows particle size distribution. **B.** SEM of representative exosome sample show exosomes of 80-100 nm (bottom and top arrow respectively) in size. **C.** Western blot analysis of exosome isolates as compared with cellular lysates. β-actin is used as a loading control for cell lysates. **D.** Exosome concentration in µg per ≈ 1×10^6 cells.

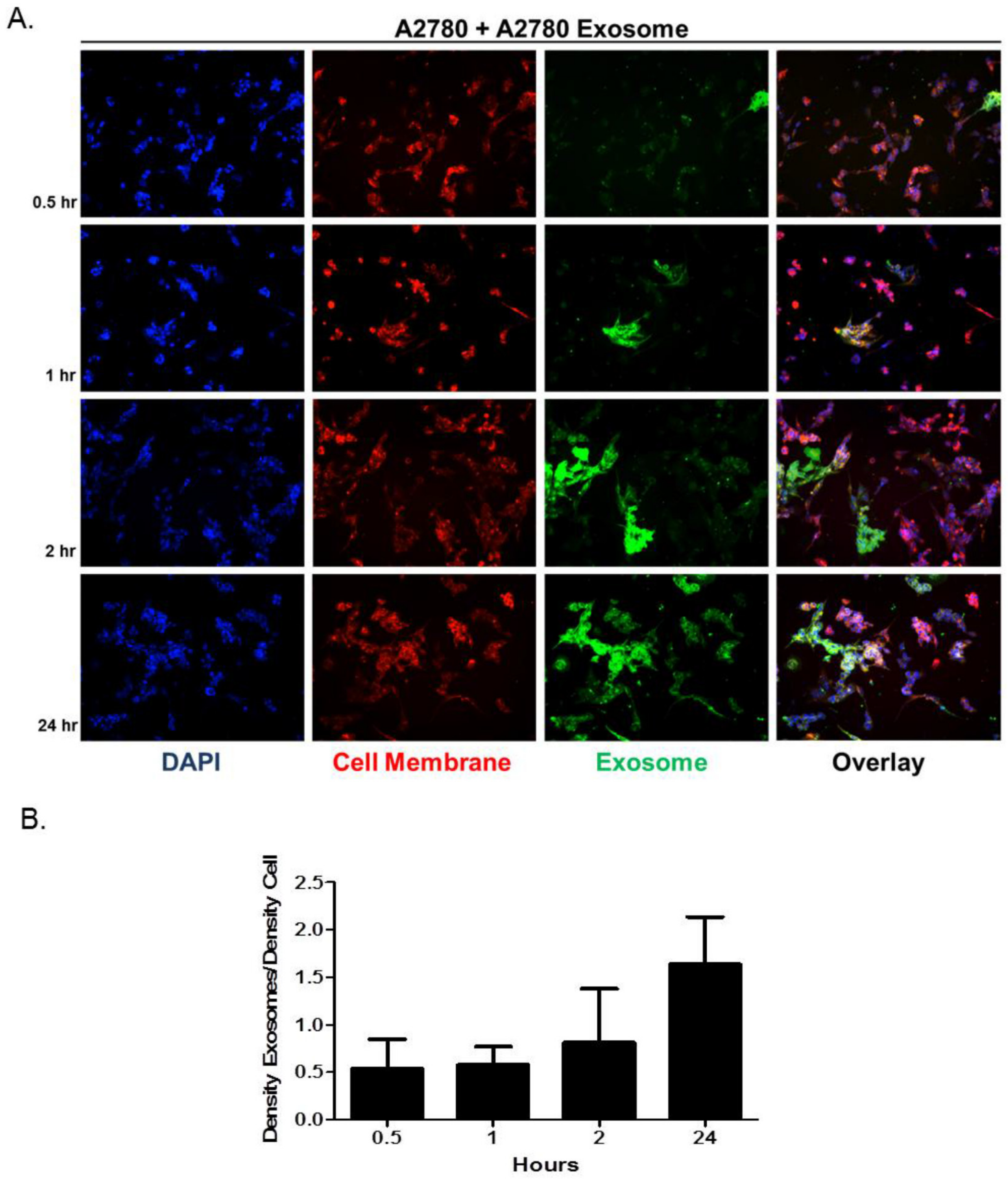

Supplementary Figure 2: A2780 Cell Uptake of A2780-derived Exosomes. A. Representative fluorescent microscopy images of dapi (blue), cell membrane (red), exogenous exosomes (green), after exosome addition at 0.5, 1, 2, and 24 hours. **B.** Average of a minimum of 3 images describing density of exosome/density of cell at each time point. Error bars represent standard error of the mean.

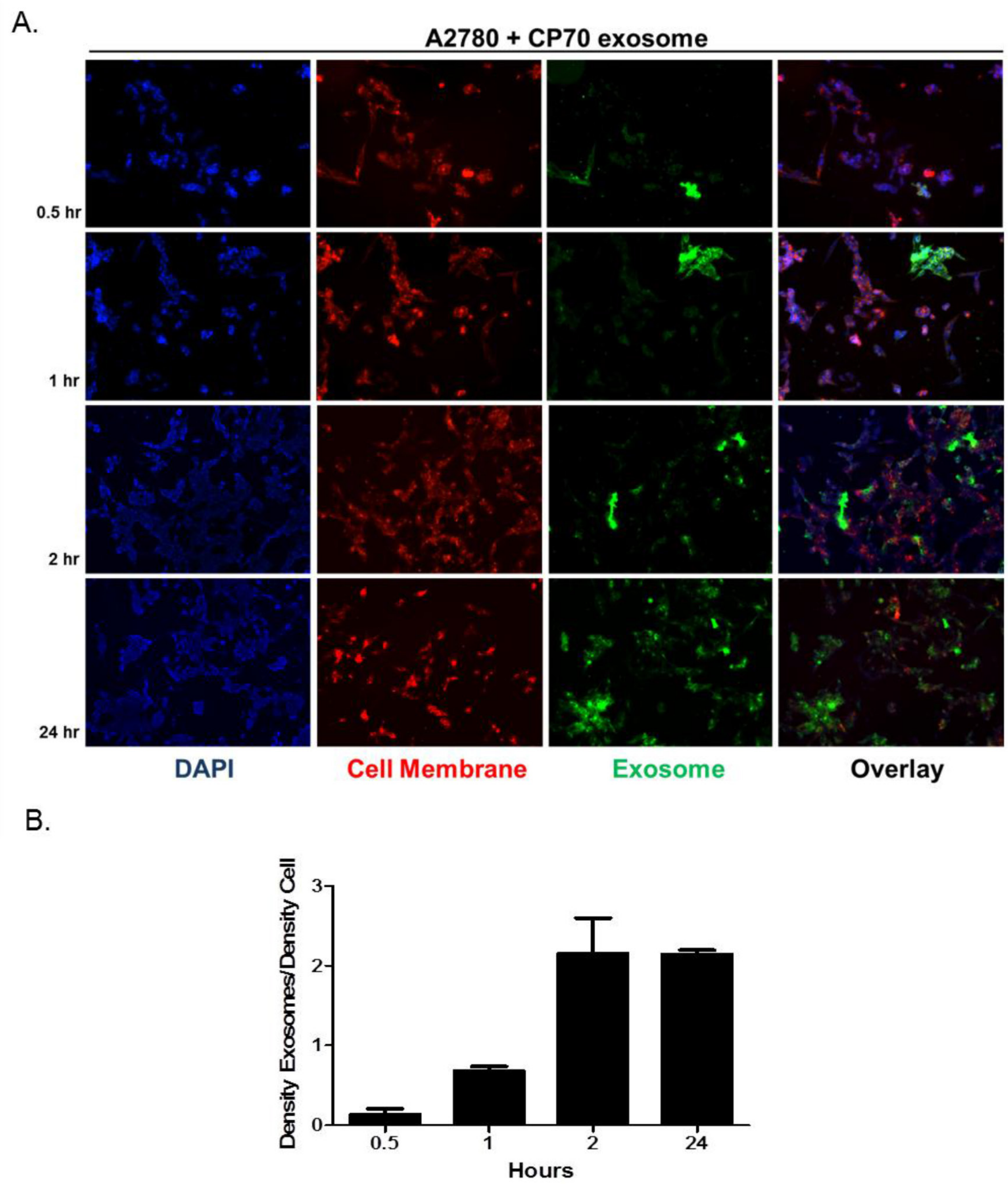

Supplementary Figure 3: A2780 Cell Uptake of CP70-derived Exosomes. A. Representative fluorescent microscopy images of dapi (blue), cell membrane (red), exogenous exosomes (green), after exosome addition at 0.5, 1, 2, and 24 hours. **B.** Average of a minimum of 3 images describing density of exosome/density of cell at each time point. Error bars represent standard error of the mean.

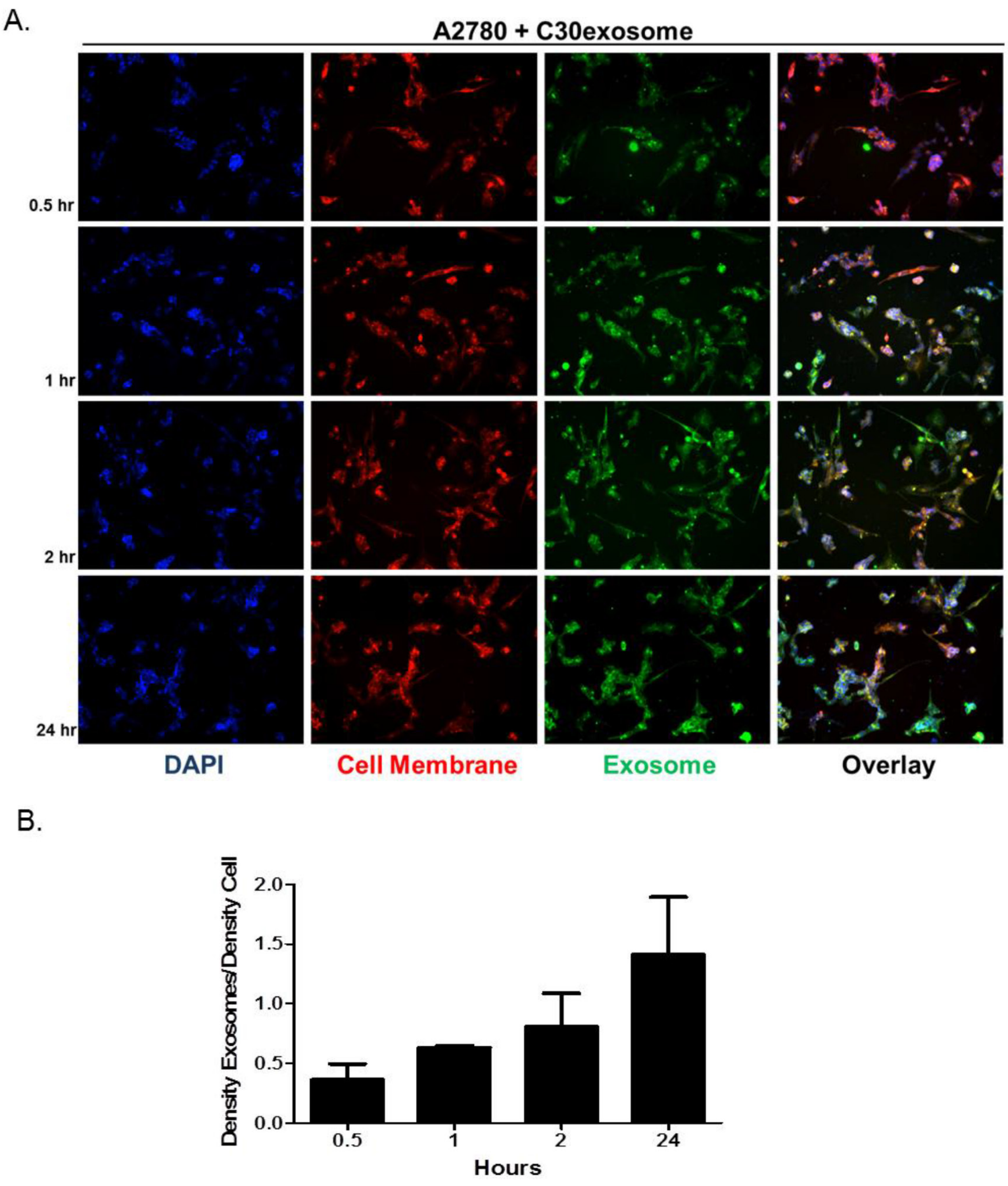

Supplementary Figure 4: A2780 Cell Uptake of C30-derived Exosomes. A. Representative fluorescent microscopy images of dapi (blue), cell membrane (red), exogenous exosomes (green), after exosome addition at 0.5, 1, 2, and 24 hours. **B.** Average of a minimum of 3 images describing density of exosome/density of cell at each time point. Error bars represent standard error of the mean.

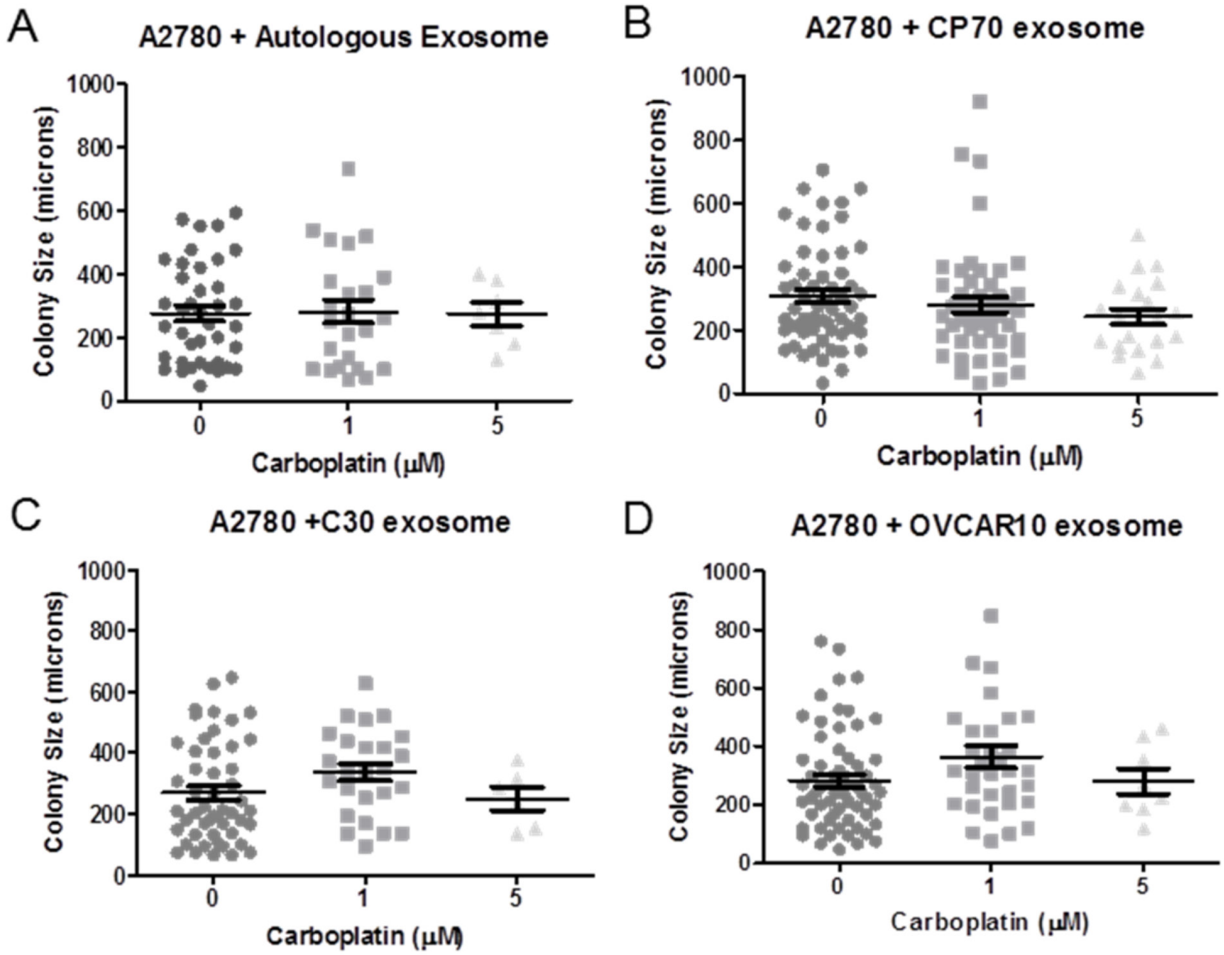

Supplementary Figure 5: Colony Formation Assays in the Presence of Carboplatin Following Pre-Treatment with Exosome. A2780 cells were pre-treated with **A.** A2780 (autologous), **B.** CP70, **C.** C30, or **D.** OVCAR10 derived exosomes, plated 100 cells per well and then grown in 0, 1, or 5 µM carboplatin for up to 3 weeks. Colony size was determined from tiff images using Adobe Photoshop measurement analysis after calibration with a micrometer assembly. Colony size is in microns.

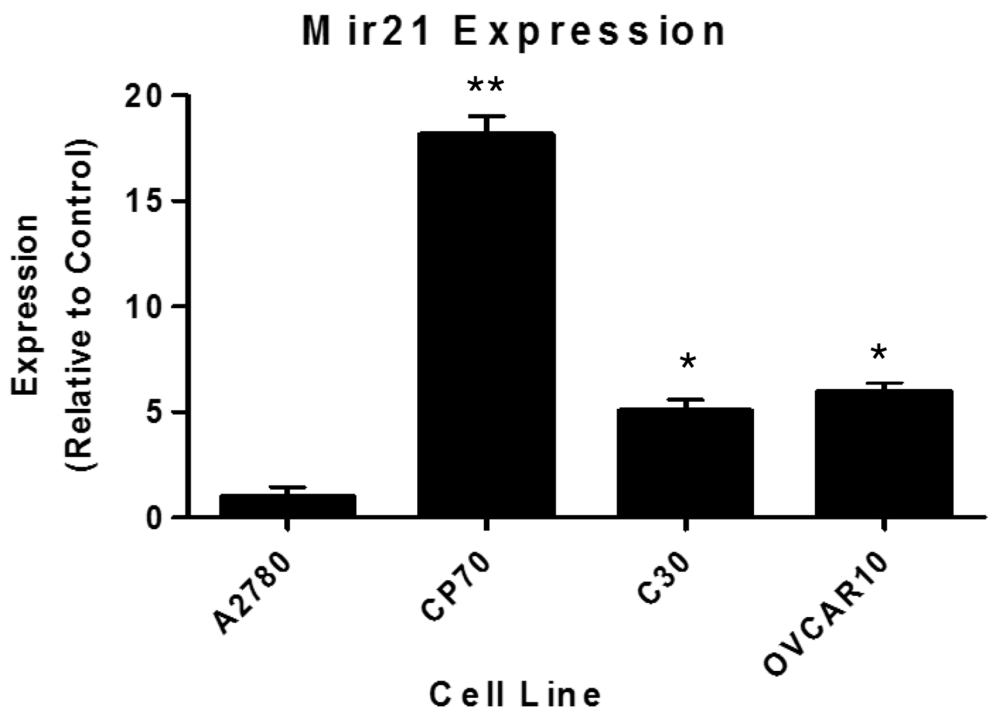

Supplementary Figure 6: Mature miR-21 Expression in EOC Cell Lines. Expression levels of miR-21 in A2780, CP70, C30, and OVCAR10 cell lines relative to U6 small RNA. Error bars represent standard error of the mean. Significant increases are compared to A2780. n=3. **p<0.01. *p<0.05.

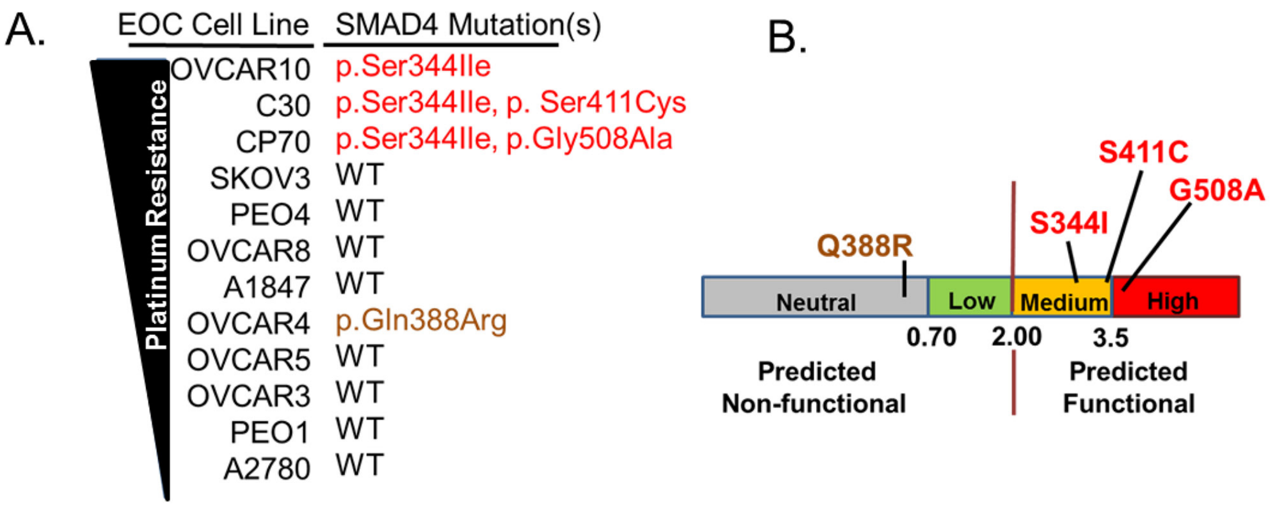

Supplementary Figure 7: Mutations in *SMAD4* are Associated with Platinum Resistance. A. Illustration of SMAD4 mutations identified in EOC cell lines ranked according to platinum-resistance (Top – most resistant, Bottom – least resistant). **B.** Functional impact scores as predicted using Mutation Assessor, which mathematically assesses functional impact based upon an algorithm combining the overall conservation of the gene with the specificity of the mutation. Values <0.70 are considered neutral, values between 0.71 and 2.0 are considered low, and values between 2.1 and 3.5 and >3.6 are considered to have medium and high impact, respectively.

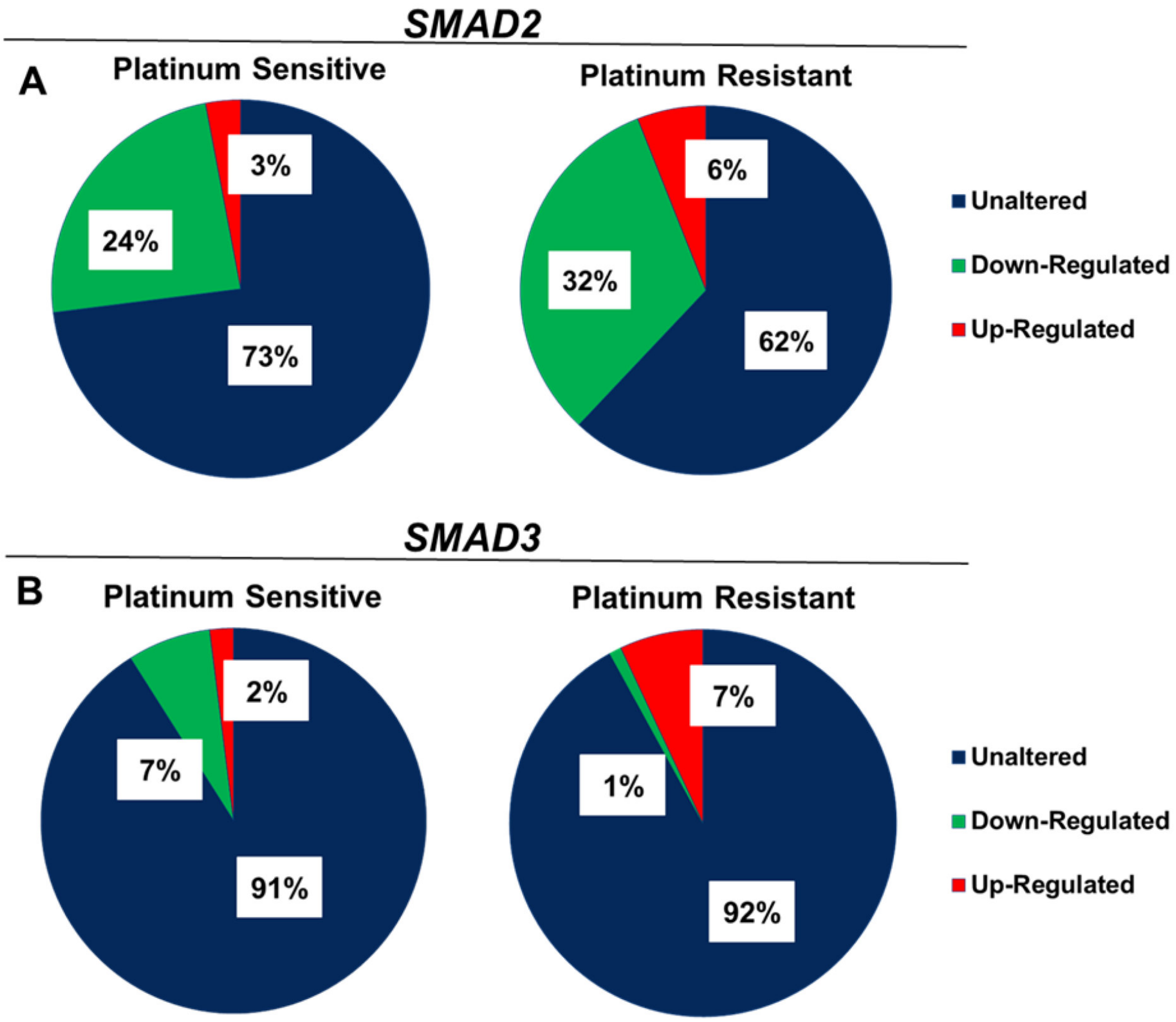

Supplementary Figure 8: Loss of *SMAD2* Expression Corresponds with a Platinum-Resistant Phenotype. A. TCGA data analyzed to show variations in regulation of *SMAD2* mRNA between platinum-resistant (n=197) and platinum-sensitive (n=90) patient tumor samples. **B.** TCGA data analyzed to show variations in regulation of *SMAD3* mRNA between platinum-resistant (n=197) and platinum-sensitive (n=90) patient tumor samples.

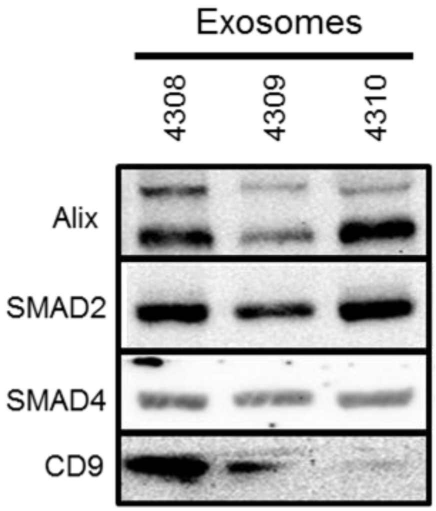

Supplementary Figure 9: SMAD2 and SMAD4 Proteins are Present in Exosomes Isolated from Blood Samples from Healthy Individuals. Western blot analysis of SMAD4 and SMAD2 in exosomes isolated from healthy human plasma, n=3. Alix and CD9 are exosomal markers.

Supplementary Table 1: EOC Cell Line Carboplatin IC_{50}s

Cell Line	IC_{50} (µM)	Fold Change
A2780	11	1
OVCAR5	44	4
A1847	67	6
CP70	120	11
OVCAR10	200	18
C30	325	30
C200	>500	>50

IC_{50} values of the cell lines used in this study are listed above. A2780 cells exhibit the most sensitivity to carboplatin. OVCAR5 and A1847 cell lines are also considered sensitive with 4 and 6-fold increases in resistance respectively as compared to A2780. CP70, OVCAR10, C30 and C200 are considered the most resistant with 11,18, 30, and >50-fold increases in resistance respectively, as compared to A2780.

Supplementary Table 2: Genes in the TruSeq Amplicon – Cancer Panel

ABL1	EGFR	GNAS	MLH1	RET
AKT1	ERBB2	HNF1A	MPL	SMAD4
ALK	ERBB4	HRAS	NOTCH1	SMARCB1
APC	FBXW7	IDH1	NPM1	SMO
ATM	FGFR	JAK2	NRAS	SRC
BRAF	FGFR2	JAK3	PDGFRA	STK11
CDH1	FGFR3	KDR	PIK3CA	TP53
CDKN2A	FLT3	KIT	PTEN	VHL
CSF1R	GNA11	KRAS	PTPN11	
CTNNB1	GNAQ	MET	RB1	

Supplementary Table 3: Primary Antibodies and Sources

Primary Antibody	Source
Anti-TSG101-(clone C2)<br>Anti-ALIX (clone 3A9)<br>Anti-GRP78 (clone G-10)	Santa Cruz Biotechnology
Anti-β-actin (clone AC-74)	Sigma Aldrich
Anti-SMAD4<br>Anti-SMAD2 (clone 86F7)<br>Anti-PDCD4 (clone D29C6 XP®)<br>Anti-ZEB (clone D80D3)<br>Anti-N-cadherin	Cell Signaling
Anti-TGFβrI (lot 2344723)<br>Anti-TGFβrII (Lot 2283846)	Millipore
